# Genome-wide characterization of *WRKY* family genes in four araceae species and their expression analysis in *Amorphophallus konjac*

**DOI:** 10.3389/fpls.2025.1671100

**Published:** 2025-12-12

**Authors:** Haoliang Shi, Ying Zou, Min Yang, Ying Qi, Penghua Gao, Yongteng Zhao, Feiyan Huang, Jiani Liu, Jianrong Zhao, Lifang Li, Lei Yu

**Affiliations:** Yunnan Key Laboratory of Konjac Biology, College of Agronomy, Yunnan Urban Agricultural Engineering and Technological Research Center, Kunming University, Kunming, China

**Keywords:** *WRKY*, araceae, *Amorphophallus konjac*, biotic and abiotic stresses, gene expression profiling

## Abstract

**Introduction:**

The Araceae family is a large family of angiosperms containing many economically valuable and ecologically important species, such as *Amorphophallus*, *Zantedeschia elliottiana*, and *Spirodela intermedia*. The *WRKY* family is one of the largest plant-specific transcription factor families and plays a crucial role in plant responses to biotic and abiotic stresses.

**Methods:**

In this study, WRKY family members were identified and characterized in four species—*Amorphophallus konjac, Amorphophallus albus, Zantedeschia elliottiana*, and *Spirodela intermedia*—using bioinformatics approaches. Characterization included analyses of physicochemical properties, gene structure, phylogenetic relationships, chromosomal distribution, collinearity, and cis-regulatory elements. Expressions were specifically performed in *A. konjac* using transcriptomics data to examine AkWRKY expression across various tissues and stages of corm development. These expression profiles were further validated by quantitative real-time PCR (qRT-PCR), including tissue types (leaf, petiole, corm, and root); hormone treatments (abscisic acid (ABA)); jasmonic acid (JA); salicylic acid (SA); biotic stress (infection by *Pectobacterium carotovorum* subsp. *carotovorum* (Pcc)), and abiotic stresses (low temperature, drought, and salt).

**Results:**

A total of 79, 57, 59, and 36 *WRKY* members were identified in *A. konjac*, *A. albus*, *Z. elliottiana*, and *S. intermedia*, respectively, with the majority predicted to be localized in the nucleus. Most *WRKY* members contained the conserved heptapeptide WRKYGQK domain within their motifs, and genes within the same subgroup shared similar gene structures and motif distributions. Phylogenetic analysis revealed that most Araceae *WRKY* members belong to Group II. Collinearity analysis indicated that segmental duplication was the primary driving force for the expansion of the *WRKY* gene family in these Araceae species (Ka/Ks < 1), suggesting the action of purifying selection. Cis-element analysis revealed that the promoter regions of *WRKY* genes contain numerous regulatory elements associated with plant growth and development, hormone regulation, stress responses, and light responses. Transcriptome analysis demonstrated that *AkWRKYs* exhibit tissue-specific expression patterns in leaves, petioles, corms, and roots, with most genes revealing up-regulated expression during developmental stages 2 to 3 of the corm. To elucidate the expression patterns of *AkWRKYs* under biotic and abiotic stresses, qRT-PCR was used to analyze the expression profiles of 14 *AkWRKYs* in response to ABA, JA, SA treatments, *Pcc* infection, as well as low temperature, drought, and salt stress. These 14 *AkWRKY* members displayed significantly differential expression characteristics under hormone regulation, biotic stress, and abiotic stress, responding to various stress treatments to different degrees over time.

**Conclusion:**

Among the 79 identified *AkWRKY* members, *AkWRKY38* and *53* exhibited high expression levels in *A. konjac* under hormone treatments, biotic stress (*Pcc* infection), and abiotic stresses (low temperature, drought, and salt stress). This study provided new insights into the roles of *WRKYs* in *A. konjac* responses to soft rot disease, low temperature, drought, and salt stress. Additionally, it laid a foundation for breeding stress-resistant *A. konjac* cultivars.

## 1 Introduction

Throughout a plant’s life cycle, it regularly encounters types of stress that can severely hinder optimal growth and significantly reduce yield (Garbeva and Weisskopf, 2020). To defend against or adapt to these different stresses, plants have evolved a range of regulatory mechanisms, including extensive regulation of numerous genes that mediate physiological and biochemical processes (Singh et al., 2002). Transcription factors (TFs) are crucial proteins that bind to specific DNA motifs to regulate gene expression and play key roles in plant growth, development, metabolism, and stress response (Ryu et al., 2006). WRKY TFs are one of the largest and most widely studied families of transcriptional regulators in higher plants (Singh et al., 2002; Ulker and Somssich, 2004; Ryu et al., 2006; Garbeva and Weisskopf, 2020). Since the cloning of the first *WRKY* gene (*SPF1*) in sweet potato (Ishiguro and Nakamura, 1994), *WRKY* genes have been identified in a wide range of species, including arabidopsis (72 genes) (Dong et al., 2003), rice (103) (Ross et al., 2007), maize (120) (Zhang et al., 2017), and cucumber (61) (Chen et al., 2020). The main defining feature of WRKY proteins is their *WRKY* domain, which includes a conserved heptapeptide sequence, WRKYGQK, at the N-terminal end, and a C2H2 or C2HC zinc finger structure of approximately 60 amino acids at the C-terminal end (Rushton et al., 2010). Based on the number of *WRKY* domains and the characterization of their zinc finger structures, WRKY proteins are classified into three major groups: I, II, and III. Group I includes proteins with two *WRKY* domains and a C2H2 zinc finger motif. Group II comprises proteins with a single *WRKY* domain and a C2H2 motif and is further categorized into five subgroups: IIa, IIb, IIc, IId, and IIe. Group III includes proteins that contain a single *WRKY* domain and C2HC zinc finger motif (Ling et al., 2011; Phukan et al., 2016). The transcriptional regulatory functions of *WRKY* are primarily dependent on nuclear localization signals, leucine zippers, and amino acid-enriched regions, including serine or threonine, glutamine, proline, and kinase structural domains (Jiang et al., 2024).

The WRKY family of TFs is involved in many aspects of plant growth and development (Rushton et al., 2010), including the regulation of seed dormancy (Ding et al., 2014), flowering (Li et al., 2016), male gametogenesis (Lei et al., 2017), leaf senescence (Gu et al., 2019), trichome development (Pesch et al., 2014), and the positive regulation of crop leaf angle to improve yields (Gu et al., 2019). WRKY TFs also serve as key regulators of secondary metabolic pathways, modulating the biosynthesis and accumulation of secondary metabolites (Liu L. et al., 2024), such as artemisinin, alkaloids, phenylpropanols, and anthocyanins (Chen et al., 2017; Javed and Gao, 2023). In addition, WRKY TFs play a crucial role in signaling and gene expression regulation during responses to both biotic and abiotic stresses. In response to biotic stresses, WRKY transcription factors play diverse regulatory roles across plant species. In chrysanthemum (*Chrysanthemum morifolium*), the interaction between the *CmWRKY-15–1* and the *CmNPR1* activates the downstream expression of *PR1*, *PR2*, and *PR10*, enhancing resistance to *Trichoderma harzianum* infection (Gao G. et al., 2022). In cotton (*Gossypium hirsutum*), the *GhWRKY70* negatively regulates defense against dahlia yellow wilt by up-regulating the expression of *PR1* and *NPR1*, both associated with the salicylic acid (SA) signaling pathway (Xiong et al., 2019). In soybean (*Glycine max*), the *GmWRKY40* is strongly induced following soybean blast infestation, and its silencing increases the plant’s susceptibility to the soybean blast fungus (Cui et al., 2019). In response to abiotic stresses, various *WRKYs* exhibit differential expression across plant species. In flax (*Camelina sativa* L.) *CsWRKY21* is highly expressed under low-temperature stress, whereas *CsWRKY22* exhibits high expression under drought stress (Song et al., 2020). In cucumber (*Cucumis sativus* L.), five *CsWRKYs* respond strongly to salt and high-temperature stresses (Chen et al., 2020). Similarly, in sugarcane (*Saccharum officinarum* L.), five *CsWRKYs* are highly expressed under salt and high-temperature conditions. The expression of the *ScWRKY3* increases in response to salt, polyethylene glycol (PEG), and abscisic acid (ABA) treatments but decreases under SA and jasmonic acid (JA) treatments (Wang et al., 2018). Additionally, the *ScWRKY5* is regulated by salt, PEG, SA, and ABA treatments (Wang et al., 2020).

The Araceae family is a significant group of angiosperms that contains numerous species with unique ecological adaptations and economic values. Recently, as phylogenetic and genomic studies of Araceae have deepened, key events in this family’s evolutionary history—such as genome-wide duplication events—have been revealed (Zhao et al., 2023). These genome-level changes may have displayed a critical impact on the evolution and functional differentiation of the *WRKY* family. *Amorphophallus konjac*, *Amorphophallus albus*, *Zantedeschia elliottiana*, and *Spirodela intermedia* are notable members of the Araceae family, exhibiting significant differences in morphology, ecological habits, and economic uses. *A. konjac* and *A. albus*, both belonging to the genus *Amorphophallus*, are widely distributed across Southeast Asia and Southwest China (Zhao et al., 2023). Their underground corms are rich in Konjac glucomannan, which is used as a thickener, stabilizer, and gelling agent in the food processing, biotechnology, and pharmaceutical industries (Devaraj et al., 2019). In the medical field, glucomannan is also known for its ability to lower blood lipids and glucose levels, support metabolic regulation, and contribute to the development of functional foods and health products (Wang et al., 2021). Konjac corms are also rich in alkaloids (Liu, 2004; Hu et al., 2025), which possess certain antibacterial properties (Das et al., 2010). However, the expansion of cultivation area, irrational continuous cropping practice, insufficient preventive measures, and worsening environmental pollution have become major factors restricting the high-quality development of the konjac industry. Key challenges include cold damage caused by extreme weather, drought resulting from water scarcity, irrigation water pollution due to improper management, and an increased incidence of soft rot disease in konjac caused by the increase of pathogenic bacteria in the soil (Sharma et al., 2019; Li et al., 2023). *A. konjac* has a larger tuber, high yield, low planting cost, broad adaptability, and a more developed industrial chain, making it one of the most widely cultivated konjac species in China (Jain et al., 2025). *Z. elliottiana* has gained prominence in the ornamental flower market due to its distinctive yellow spathe (Wang et al., 2023), whereas *S. intermedia*, a small aquatic plant, is used for fodder, food, fuel, and wastewater remediation (Hoang et al., 2020). However, the identification and functional analysis of *WRKY* family members in these four plant species have not yet been reported.

In this study, we screened members of the WRKY TF family in *A. konjac*, *A. albus*, *Z. elliottiana*, and *S. intermedia* for the first time using whole-genome data. We then analyzed their physicochemical properties, phylogenetic relationships, conserved motifs and domains, gene structures, collinearity, and protein–protein interaction networks using bioinformatics methods. Transcriptome data were used to analyze the differential expression of WRKY TFs during the four stages of *A. konjac* bulb development. Quantitative real-time PCR (qRT-PCR) was employed to assess the expression patterns of the *AkWRKY* family members across various tissues (root, petiole, corm, and leaf) and under both biotic stress (infection with *Pectobacterium carotovorum* subsp. *Carotovorum* (*Pcc*)) and abiotic stress (ABA, JA, SA, low temperature, drought, and salt) treatments. The findings provide a theoretical foundation for further investigation into WRKY TFs involved in the resistance mechanism in *A. konjac*, as well as a genetic resource for the improvement of disease-resistant *A. konjac* varieties.

## 2 Materials and methods

### 2.1 Identification and physicochemical characterization of *WRKY* family members in Araceae

Genomic assembly data of *A. konjac* (GCA_022559845.1) (Gao Y. et al., 2022) and *A. albus* (GCA_047678385.1) (Duan et al., 2025), along with the genomic and annotation data of *S. intermedia* (GCA_902703425.1) (Hoang et al., 2020), were obtained from NCBI. The annotation files of *A. konjac* (https://doi.org/10.6084/m9.figshare.15169578) and *A. albus* (https://doi.org/10.6084/m9.figshare.15169578), as well as the genomic and annotation files of *Z. elliottiana* (10.6084/m9.figshare.22656112), were downloaded from Figshare. The data for *Arabidopsis thaliana* 72 *AtWRKY* family members were downloaded from the TAIR database (https://www.arabidopsis.org/). Members of *WRKYs* were screened in the *A. konjac*, *A. albus*, *Z. elliottiana*, and *S. intermedia* genome databases using the BLASTp (E-value < 10^-5^) function in the TBtools software (Chen C. et al., 2023). Subsequently, we downloaded the *WRKY* Hidden Markov Model (PF03106) from the Pfam database (https://pfam.xfam.org/) (Punta et al., 2012) and used the HMM function (E-value < 10^-5^) in TBtools to search the *A. konjac*, *A. albus*, *Z. elliottiana*, and *S. intermedia* genome databases. To ensure high-confidence identification, only sequences detected by both methods were retained as final candidate *WRKY* genes for downstream analyses.​ Typical structural domains were then analyzed using the NCBI-CDD database (https://www.ncbi.nlm.nih.gov/Structure/cdd/wrpsb.cgi) to eliminate proteins that did not contain *WRKY* structural domains and to finalize the *WRKY* family members of *A. konjac*, *A. albus*, *Z. elliottiana*, and *S. intermedia*. Physicochemical properties, such as amino acid content, CDS length, molecular weight, isoelectric point, aliphatic amino acid index, and the hydrophobicity index of proteins were analyzed using the ProtParam tool in TBtools. The online tool WoLF PSORT (https://wolfpsort.hgc.jp/) (Horton et al., 2007) was used to perform subcellular localization. The chromosomal locations of the genes were extracted from the GFF annotation files of *A. konjac*, *A. albus*, *Z. elliottiana*, and *S. intermedia* and visualized using TBtools software.

### 2.2 Phylogenetic analysis of *WRKY* family members in Araceae

To fully explore the evolutionary relationship of the *WRKY* family in the four species, multiple sequence comparison of WRKY protein sequences from *A. thaliana*, *Oryza sativa* (Khan et al., 2022), *A. konjac*, *A. albus*, *Z. elliottiana*, and *S. intermedia* was performed using MAFFT (version 7.427, –auto) (Katoh et al., 2002). Subsequently, phylogenetic analysis was performed using IQ-TREE (v1.6.10) with the raw alignment. The best-fit substitution model was selected via ModelFinder (Kalyaanamoorthy et al., 2017), with JTT+R7 identified as optimal. Maximum likelihood trees were constructed using this model with 1000 ultrafast bootstrap replicates (-bb 1000) (Nguyen et al., 2015). In addition, the phylogenetic tree was rooted using the Minimal Ancestor Deviation method (MAD; implemented by the ‘Root a PhyloTree’ plugin in TBtools) (Tria et al., 2017; Abrusán and Zelezniak, 2024). This method was selected because it algorithmically determines the root position that minimizes root-to-tip distance variance, thereby providing an objective rooting criterion. The MAD approach is especially valuable for our dataset, as these sequences lack an appropriate, distantly related outgroup. Finally, the evolutionary tree was visualized and beautified using the online tool iTOL (version 6) (https://itol.embl.de/) (Letunic and Bork, 2021). The original unrooted tree, with clades colored consistently with the main figures, is shown in **Supplementary Figure S1**.

### 2.3 Analysis of conserved motifs, structural domains, and gene structures of *WRKY* family members in Araceae

The MEME function in TBtools software was used to independently identify the conserved motifs of the *WRKY* family in the four species, and the maximum number of motifs was set to 10, and all other parameters were left at their default values. TBtools software was also used to determine the introns or exons distribution of the *WRKY* family in the four species according to their genome annotation files. Finally, TBtools software was used to construct maps of gene structure, conserved structural domains, and conserved motif combinations (Chen C. et al., 2023).

### 2.4 Analysis of cis-acting elements in the promoters of *WRKY* family members in Araceae

The 2000 bp upstream promoter sequences of the *WRKYs* of the four species were extracted separately using TBtools software and submitted to the PlantCARE website (http://bioinformatics.psb.ugent.be/webtools/plantcare/html/) (Lescot et al., 2002) for cis-acting element prediction. The results were organized using Microsoft Excel software and then visualized with TBtools.

### 2.5 Intraspecies and interspecies collinearity analysis and Ka/Ks analysis of *WRKY* family members in Araceae

Intraspecies collinearity analyses of *A. konjac*, *A. albus*, *Z. elliottiana*, and *S. intermedia* were performed using the MCScanX (Wang et al., 2012) function in TBtools software. In addition, intraspecies collinearity analyses were conducted between *A. konjac* and each of *A. thaliana*, *A. albus*, *Z. elliottiana*, and *S. intermedia*. Ka/Ks ratios for segmental duplicate gene pairs in *A. konjac*, *A. albus*, *Z. elliottiana*, and *S. intermedia* were calculated using the Simple Ka/Ks Calculator (NG) program (Chen C. et al., 2023).

### 2.6 *AkWRKYs* protein interaction network analysis, gene ontology, and Kyoto encyclopedia of genes and genomes pathway enrichment analysis

The rich protein interaction data provided by the STRING database (https://cn.string-db.org/) (Szklarczyk et al., 2023) were used to explore the functional associations between proteins. A confidence threshold of 0.7 was set to ensure high reliability of the selected protein interactions. However, the number of interacting proteins was limited to 20 to improve the credibility and accuracy of the analysis. The interaction network AkWRKYs family protein was constructed using *A. thaliana* as the reference species model. Simultaneously, functional enrichment analysis of *AkWRKY* members was performed using GO and KEGG functions in TBtools.

### 2.7 Expression pattern analysis of *AkWRKY* family members at different developmental stages

Using the transcriptome data of *A. konjac* published by previous authors (PRJNA734512 (Gao Y. et al., 2022) and PRJNA608095 (Li et al., 2023)) to extract the expression information of target genes from different tissues and four developmental stages of *A. konjac* corms. Visualized the expression data of *AkWRKYs* genes using the HeatMap program in TBtools software (Chen C. et al., 2023).

### 2.8 Stress treatment and sampling for AkWRKY expression

The *A. konjac* plants used in this study were obtained from Yunnan Provincial Urban Agriculture Engineering and Technology Research Center/Yunnan Provincial Key Laboratory of Konjac Biology. Roots, petioles, corms, and leaves of normally growing *A. konjac* plants (2 weeks of age) were collected and stored at –80°C for later use. *A. konjac* plants in good condition and completely healthy after two months of greenhouse growth were selected, and 100 μL (1 × 10^8^ CFU/mL) of *Pectobacterium carotovorum* subsp. *carotovorum* EccK-23B (*Pcc* (MN653919)) bacterial suspension was inoculated into the petioles. Based on the symptom of *Pcc* infestation, samples were collected at four time points: Before inoculation (control (CK)), and at 24, 48, and 72 h after inoculation (individual plants were selected for each sampling). The control (CK) group was inoculated with an equal volume of sterile water, while all other environmental conditions (including light intensity, relative humidity, irrigation, and fertilization regimes) were maintained identical to those in the pathogen-treated group. Samples were collected simultaneously, with one sample taken at each inoculation time point. Each treatment included three biological replicates, with each replicate consisting of three *A. konjac* plants. Spreading leaves of *A. konjac* were subjected to low temperature, drought, hormone, and salt stress treatments. Low temperature stress was applied at 4°C, and drought stress was induced using 200 mM mannitol for 24 and 48 h. Hormone treatments involved exogenous spraying with 100 μM ABA, JA, and SA, respectively. Salt stress was applied using 200 mM NaCl. Samples were collected from the spreading leaves at 24 and 48 h after each treatment. The samples were stored at –80°C. The samples were stored at –80°C. All samples were prepared with three biological replicates.

### 2.9 RNA extraction, reverse transcription, and qRT-PCR

Total RNA was extracted from the *A. konjac* samples according to the instructions of the RNAex Pro RNA Extraction Kit (Takara). RNA degradation and contamination were monitored on a 1% agarose gel. RNA purity was assayed using a NanoPhotometer^®^ spectrophotometer (IMPLEN, California, USA). The RNA solution obtained in the previous step was reverse transcribed into a cDNA solution following the instructions of the Evo M-MLV Reverse Transcription Kit (Takara). qRT-PCR was performed using the SYBR Green Pro Taq HS Fluorescence Quantification Kit (Takara). The 20 μL reaction mixture contained 10 μL of ArtiCanCEO SYBR qPCR Mix, 0.8 μL of Primer F, 0.8 μL of Primer R, 1 μL of Template (cDNA), and 7.4 μL of ddH_2_O. The amplification program was set as follows: 95°C for 15 s; 60°C for 20 s, and 72°C for 20 s, for a total of 40 cycles. Three biological replicates were performed for each sample. Primers were as presented in **Supplementary Table S1**. The experimental data were analyzed using the relative quantitative 2^–ΔΔCt^ method (Schmittgen and Livak, 2008). One-way nested analysis of variance was performed using IBM SPSS statistical software. Bar graphs were plotted using Origin Pro 2021 software.

## 3 Results and analysis

### 3.1 Identification and physicochemical properties of *WRKY* family members in Araceae

**Figure 1:**
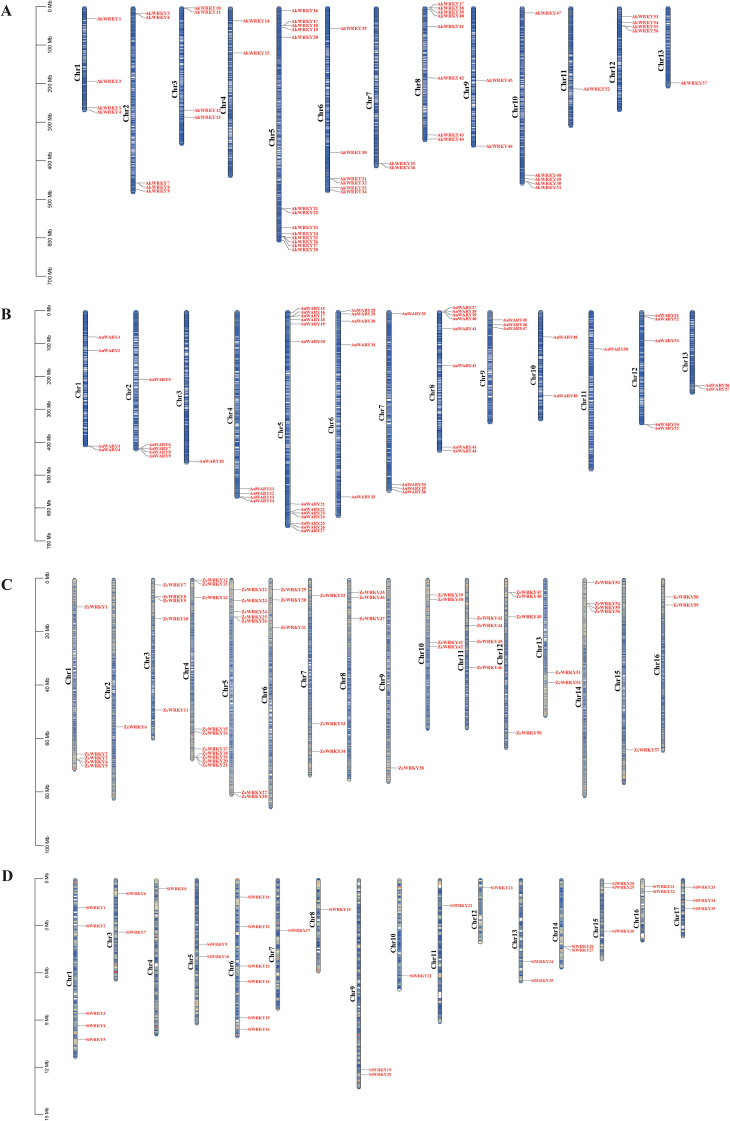
The chromosomal location and distribution of *WRKY* gene family members on chromosomal maps across four species in the Araceae family: **(A)***AkWRKY* gene, **(B)***AaWRKY* gene, **(C)***ZeWRKY* gene, **(D)***SiWRKY* gene. The scale bar represents megabases (Mb).

The results of the genome-wide searches using BLAST comparison and Hidden Markov Modeling in *A. konjac*, *A. albus*, *Z. elliottiana*, and *S. intermedia*, combined with conserved domains prediction, identified 79 *AkWRKYs*, 57 *AaWRKYs*, 59 *ZeWRKYs*, and 36 *SiWRKYs*, respectively (**Supplementary Table S2**). Based on their positions on the chromosomes, they were sequentially named *AkWRKY1*-*AkWRKY79*, *AaWRKY1*-*AaWRKY57*, *ZeWRKY1*-*ZeWRKY59*, and *SiWRKY1*-*SiWRKY36* (**Supplementary Table S3**, **Figure 1**). Genes were localized on all 13 chromosomes of *A. konjac*, with Chr5 harboring the highest number of genes (13) (**Figure 1A**). Consistent with *A. konjac*, *A. albus* showed gene distribution across all 13 chromosomes, with Chr5 exhibiting the highest density (13 genes) (**Figure 1B**). In *Z. elliottiana*, genes were distributed across all 16 chromosomes, with Chr4 exhibiting the maximum count of 10 genes (**Figure 1C**). For *S. intermedia*, genes were localized on 17 of its 18 chromosomes (except Chr2), and Chr6 possessed the highest number of genes (6) (**Figure 1D**). The amino acid lengths of the genes encoded by the *WRKYs* of the four species ranged from 59 (*AkWRKY73*) to 2,335 aa (*SiWRKY27*), and the molecular weight ranged from 7,129.01 (*AkWRKY73*) to 262,010.55 Da (*SiWRKY27*). Physicochemical property analysis revealed that the isoelectric point of WRKY proteins ranged from 4.29 (*ZeWRKY20*) to 12.08 (*SiWRKY35*); the instability index ranged from 40.07 (*SiWRKY18*) to 108.48 (*SiWRKY35*); and the aliphatic index ranged from 43.21 (*AkWRKY70*) to 108.78 (*SiWRKY18*). The hydrophobicity index ranged from −1.305 (*AkWRKY73*) to 0.25 (*SiWRKY18*). Subcellular localization analysis revealed that *WRKY* members were mainly localized in the nucleus (190), chloroplast (16), plasma membrane (11), cytosol (6), endoplasmic reticulum (5), mitochondrion (1), peroxisome (1), and vacuolar membrane (1) (**Supplementary Table S2**). Chromosomal localization analysis revealed that 79 *AkWRKYs* were unevenly distributed across 13 chromosomes, with 22 anchored contigs; 57 *AaWRKYs* were unevenly distributed across 13 chromosomes; 59 *ZeWRKYs* were unevenly distributed across 16 chromosomes; and 35 *SiWRKYs* were unevenly distributed across 16 chromosomes, with one gene (*SiWRKY36*) anchored to contigs (**Figure 1**).

### 3.2 Phylogenetic analysis of *WRKY* family members in Araceae

**Figure 2:**
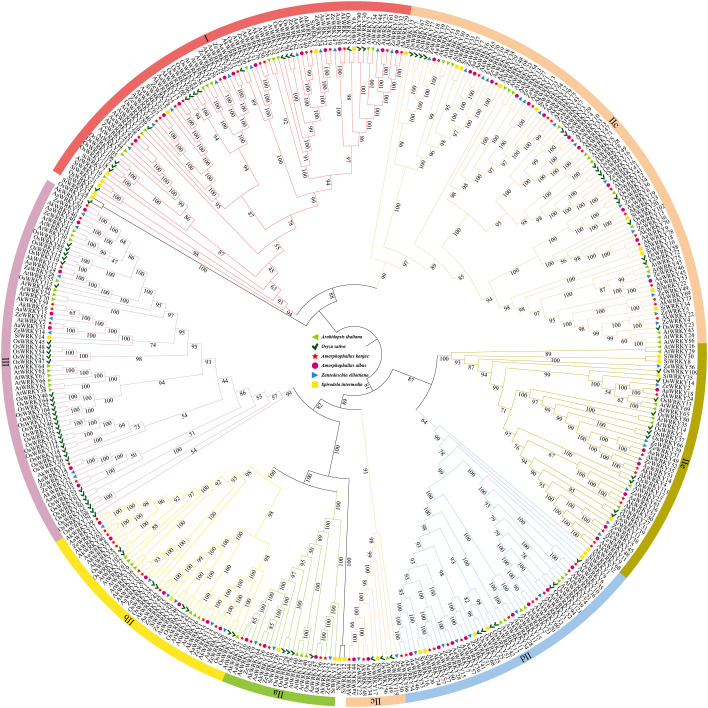
Phylogenetic relationship of *WRKY* genes in *A. thaliana*, *O. sativa*, *A. konjac*, *A. albus*, *Z. elliottiana*, and *S. intermedia* divided into 7 subfamilies, green triangles represent *A. thaliana*, dark green tick symbol represent *O. sativa*, magenta circles represent *A. konjac*, red five-pointed stars represent *A. albus*, blue triangles represent *Z. elliottiana*, and yellow squares represent *S. intermedia*.

Based on previous studies of *WRKYs* in *A. thaliana* (Wu et al., 2022; Liu et al., 2023b), *WRKY* family members in Araceae, *A. thaliana*, and *O. sativa* were categorized into three groups: I, II, and III. Group II was further divided into five subgroups: IIa, IIb, IIc, IId, IIe. Class II proteins were the most abundant type in Araceae, accounting for 62.94% of all WRKY proteins (**Figure 2**). The number and types of WRKY proteins varied between terrestrial species (*A. konjac*, *A. albus*, and *Z. elliottiana*) and the aquatic species (*S. intermedia*). In the *A. konjac*, members of Class I (16), Class II (55), amd Class III (8) were the most numerous (**Figure 2**). In Class I, three *AkWRKYs* (*AkWRKY19*, *AkWRKY62*, and *AkWRKY49*) clustered together with *AtWRKY25*, *AtWRKY26*, and *AtWRKY33*, along with *O. sativa WRKY24*, *WRKY53*, and *WRKY70* in a well-defined branch. Within Class II, four *AkWRKYs* (*AkWRKY16*, *26*, *27*, *64*) grouped with four *O. sativa WRKYs* (*WRKY28*, *62*, *71*, *76*) and three *AtWRKY*s (*AtWRKY18*, *40*, *46*) in subclade IIa, while six *AkWRKYs* (*AkWRKY28*, *54*, *57*, *60*, *66*, *74*, *77*) co-clustered with four *O. sativa WRKYs* (*WRKY25*, *42*, *51*, *68*) and two *AtWRKY*s (*AtWRKY11*, *17*) in subclade IId (**Figure 2**). *S. intermedia*, on the other hand, is relatively less abundant than the other three Araceae species in either group (**Figure 2**). There are 55 *AkWRKY* genes and 14 *AtWRKY* genes in this study that are homologous in group II (**Figure 2**).

### 3.3 Conserved motifs, conserved structural domains, and gene structure analysis of *WRKY* family members in Araceae

**Figure 3:**
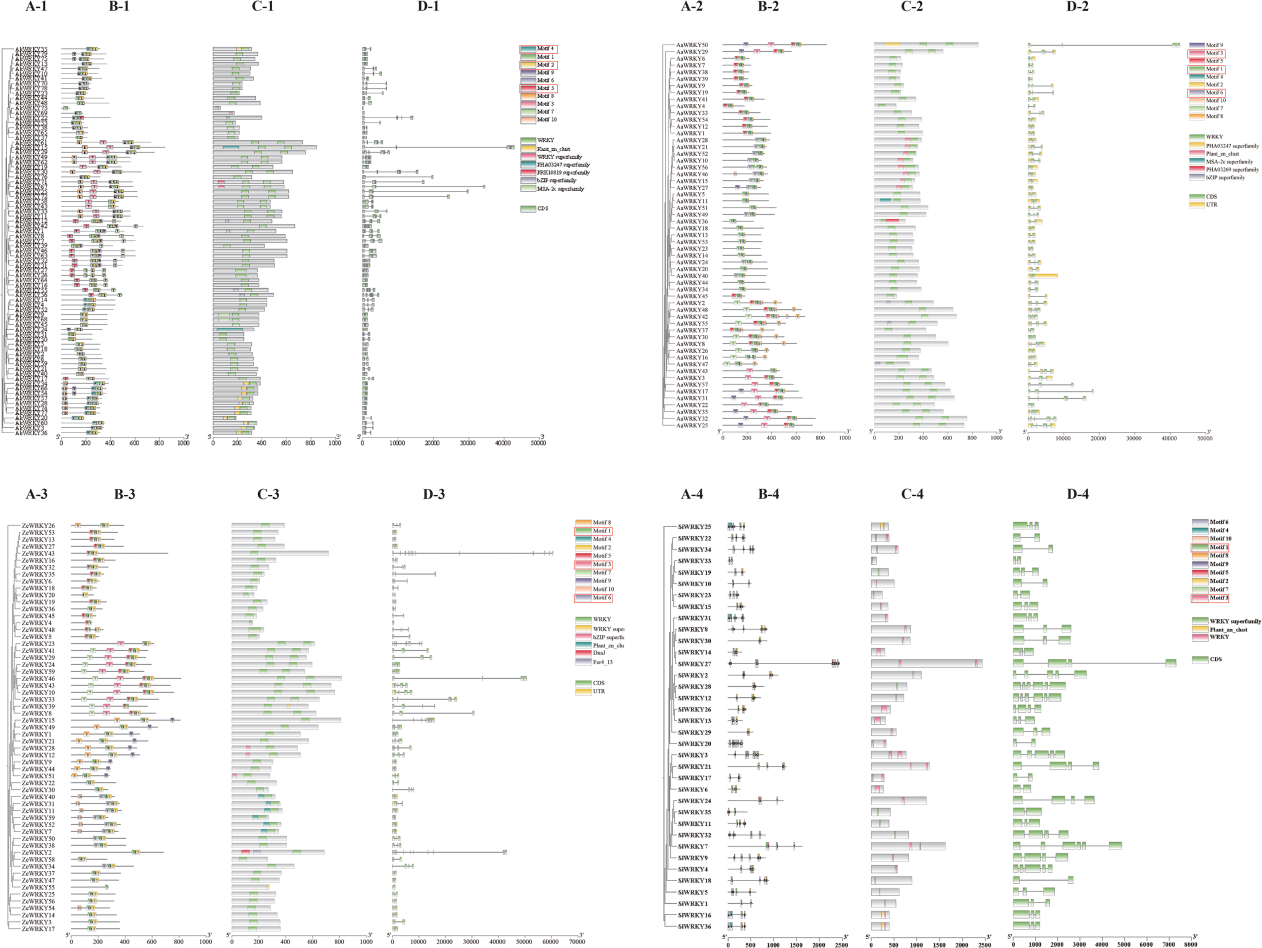
Results of **(A)** the phylogenetic tree, **(B)** the conserved motifs, **(C)** the conserved domains (the motif circled in red corresponds to the WRKY domain), and **(D)**the gene structure of WRKY gene family members in four Araceae species. Species labels: 1 (*A. konjac*), 2 (*A. albus*), 3 (*Z. elliottiana*), and 4 (*S. intermedia*).

**Figure 4:**
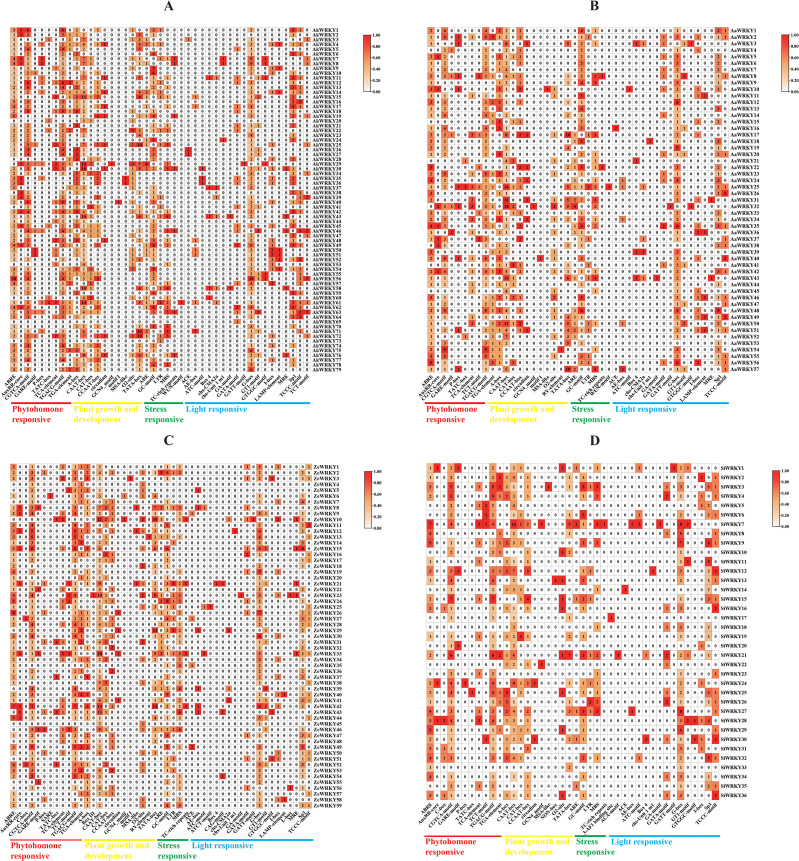
Promoter element prediction analysis of the WRKY genes across four species in the Araceae family: **(A)***AkWRKY* gene, **(B)***AaWRKY* gene, **(C)***ZeWRKY* gene, **(D)***SiWRKY* gene.

Conserved motif analysis of WRKY proteins in the four Araceae species revealed that the vast majority of *AkWRKY* members contained Motif1 and Motif2 (**Figure 3B-1**), indicating that these two motifs were relatively conserved. Motif2 and Motif4 included the *WRKY* heptapeptide structural domain, and Motif5 is a zinc finger motif (**Supplementary Figure S2A**). Most *AaWRKY* members contained Motif1, Motif2, and Motif4 (**Figure 3B-2**), suggesting that these three motifs are relatively conserved, with Motif1 and Motif3 comprising the heptapeptide domain and Motif6 representing a zinc finger motif (**Supplementary Figure S2B**). Most ZeWRKY proteins contained Motif1, Motif2, and Motif4 (**Figure 3B-3**), which are relatively more conserved compared to the three motifs. Motif1 and Motif3 include the heptapeptide structural domain, and Motif6 was identified as a zinc finger motif (**Supplementary Figure S2C**). Most *SiWRKY* members contained Motif1 (**Figure 3B-4**), suggesting that this motif is more conserved compared to the other nine. Motif1 included a heptapeptide domain, whereas Motif3 was a zinc finger motif (**Supplementary Figure S2D**). Conserved structural domain analysis revealed that all *WRKY* members contained one to three typical conserved domains (**Figures 3C-1–4**). Gene structure analysis of *WRKY* members in the four species revealed that the number of exons ranged from 1 to 7 in *AkWRKYs*, 2 to 6 in *AaWRKYs*, 1 to 15 in *ZeWRKYs*, and 2 to 6 in *SiWRKYs* (**Figures 3D-1–4**).

### 3.4 Analysis of promoter cis-acting elements of *WRKY* family members in Araceae

To further understand the transcriptional regulation of *WRKY* genes in Araceae, functional elements located 2000 bp upstream of the coding region of the four species were predicted. The results revealed that *A. konjac*, *A. albus*, *Z. elliottiana*, and *S. intermedia* contained hormone–, plant growth and development–, stress response–, and light response–related cis-acting elements. There were 9/10/6/18, 10/11/6/17,12/12/5/20, and 11/10/5/16 responsive elements in these four species, respectively, and 38 responsive elements were common to all four species. The L-box, ATCT-motif, SARE, Box III, and CAG-motif were unique to *Z. elliottiana*, whereas the ACA-motif, 3-AF1 binding site, and NON-box were unique to *S. intermedia*. Light-responsive action elements were the most abundant among all four species (**Figure 4**; **Supplementary Figure S2**). A total of 284 JA-responsive elements (CGTCA-motif and TGACG-motif) were identified in the promoters of *AkWRKYs*, along with 133 abscisic acid-responsive elements (ABRE), 31 SA-responsive elements (GARE-motif/P-box/TATC-box), 43 auxin-responsive elements (AuxRR-core/TGA-element), 29 anaerobic-inducible elements (ARE), 110 hypoxia- and flooding-inducible elements (GC-motif), 16 low-temperature–induced elements (LTR), 55 drought-induced elements (MBS), 7 defense- and stress-response elements (TC-rich repeats), and 1 wound-responsive element (WUN-motif) (**Figure 4A**). These findings suggest that *AkWRKYs* may play important roles in hormone regulation and stress responses in *A. konjac*.

### 3.5 Intraspecies and interspecies collinearity analysis and Ka/Ks analysis of *WRKY* family members in Araceae

**Figure 5:**
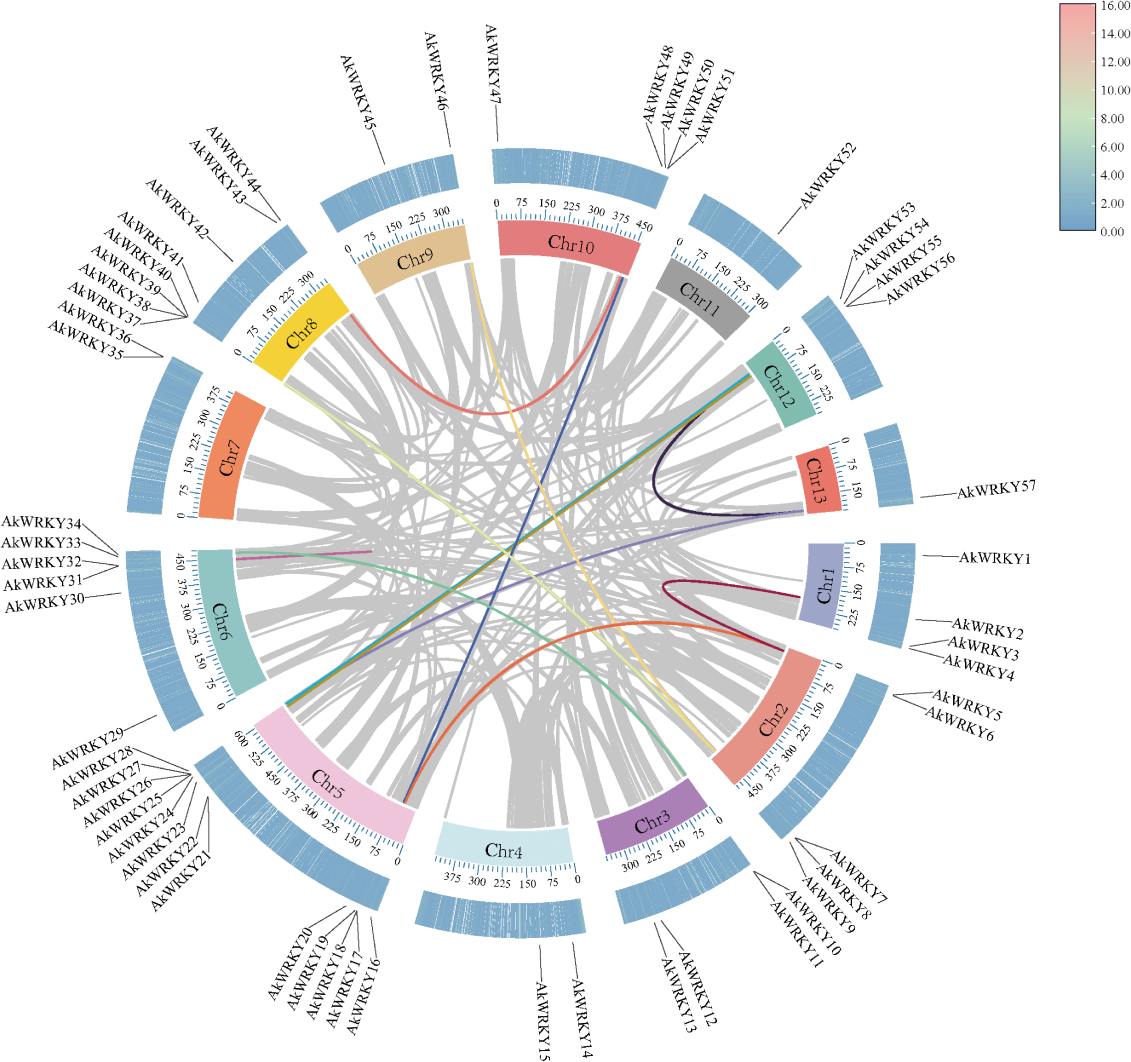
Collinearity relationships within WRKY genome across four species in the *A. konjac*, The colored lines in the middle represents the collinear relationship within the *WRKY* gene; The first circle represents the chromosome; The second circle represents the density of the *AkWRKY* gene (in heat map form).

**Figure 6:**
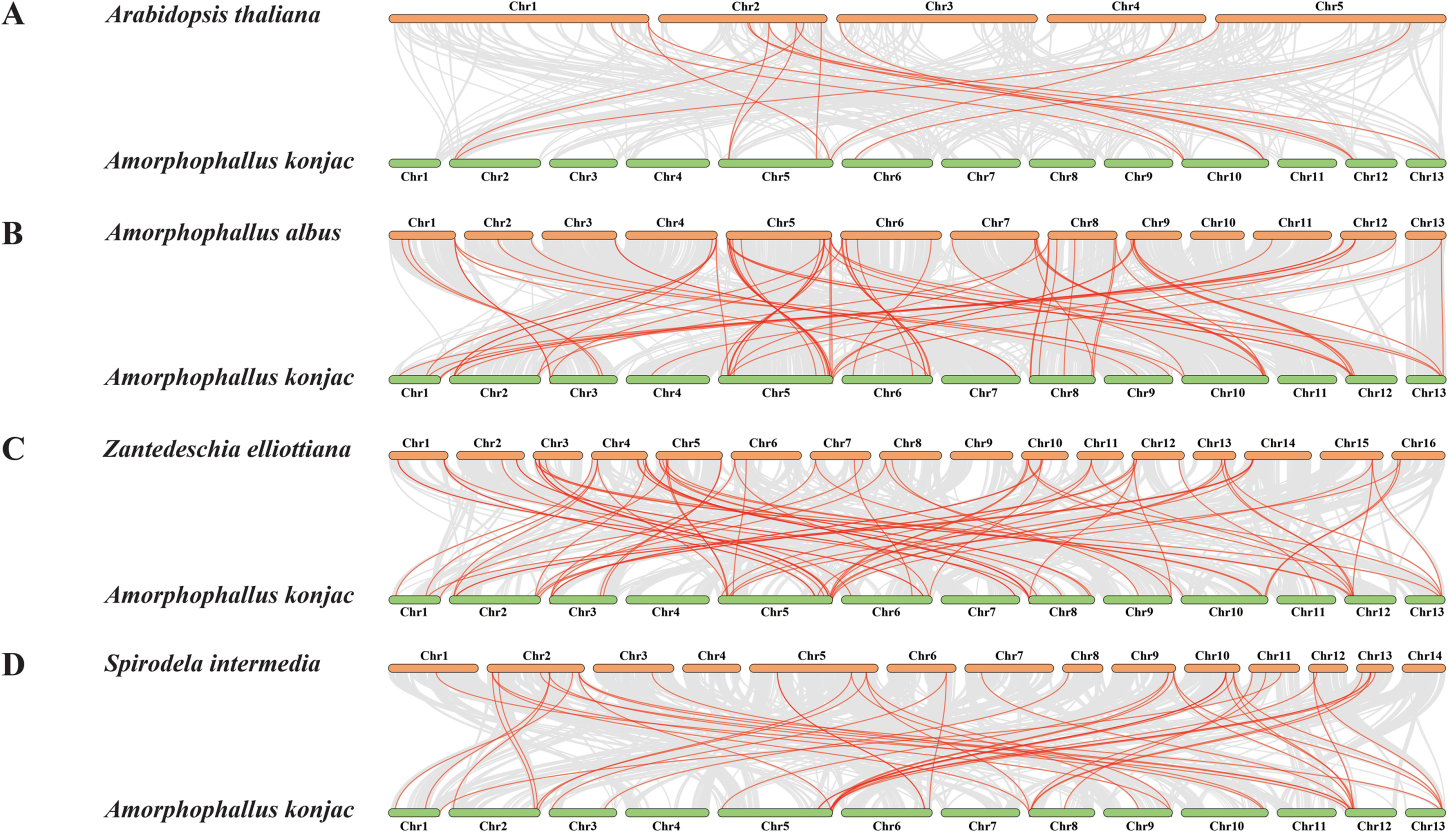
Inter-species collinearity analysis of *AkWRKY* gene family members with **(A)***A. thaliana*, **(B)***A. albus*, **(C)***Z. elliottiana*, and **(D)***S. intermedia*. The gray lines in the background represent collinear gene clusters, while the red lines indicate pairs of colinear WRKY genes with collinear relationships.

A total of 13, 18, 24, and 11 pairs of segmental duplication events involving 22, 25, 31, and 16 genes were found in *A. konjac* (**Figure 5**), *A. albus*, *Z. elliottiana*, and *S. intermedia*, respectively. Among these, the highest number of segmental duplication events occurred in *ZeWRKYs* (**Supplementary Figure S4**). To further investigate whether the *WRKYs* of the four species experienced natural selection during evolution, the Ka/Ks ratios of duplicated gene pairs were calculated. The results indicated that the Ka/Ks values for the duplicated pairs in *A. konjac*, *A. albus*, *Z. elliottiana*, and *S. intermedia* were all less than 1 (**Supplementary Table S4**), indicating that these genes have undergone purifying selection. Analysis of the interspecies collinearity of the *WRKYs* between *A. konjac* and *A. albus*, *Z. elliottiana*, and *S. intermedia* revealed that *A. konjac* formed 15 gene pairs with *A. thaliana*, 72 with *A. albus*, 77 with *Z. elliottiana*, and 48 with *S. intermedia*. Among these, *AkWRKY21*, *26*, *25*, *57*, *56*, *6*, and *49* formed covariate gene pairs between *A. konjac* and other species, suggesting that these genes have been more evolutionarily conserved (**Supplementary Table S5**; **Figure 6**).

### 3.6 Interaction network analysis of *AkWRKY* proteins and GO and KEGG enrichment analyses

The interaction network of AkWRKY proteins was constructed using the STRING online database based on comparisons with the *A. thaliana* protein database, revealing 55 nodes. Among these, the top five most connected nodes were SIB1, TIFY6A, ATG18A, QCR9, and SIB2 (**Supplementary Figure S5**). Functional enrichment analysis of 79 AkWRKY proteins based on GO annotation revealed classification into three categories: Molecular function, cellular components, and biological processes (**Supplementary Figure S6A**). Within the molecular function category, AkWRKY proteins were primarily associated with specific binding activities, including sequence-specific DNA binding, DNA-binding TF activity, heterocyclic compound binding, organocyclic compound binding, calmodulin binding, and general binding activity. Cellular component analysis revealed that most AkWRKY proteins were localized in the nucleus, consistent with their roles as TFs. In the biological process category, AkWRKY proteins were enriched in regulatory function related to metabolism and biosynthesis (including RNA/DNA metabolism and primary and secondary metabolism), as well as signaling and response pathways (involving hormones, biotic and abiotic stresses, development, and senescence). KEGG pathway analyses further revealed that AkWRKY proteins participate in categories such as environmental adaptation, organismal systems (for example, plant-pathogen interactions), signal transduction, environmental information processing, TFs, and genetic information processing (**Supplementary Figure S6B**).

### 3.7 Transcriptome expression analysis of *AkWRKYs* in different tissues of *A. konjac* and at different developmental stages of the bulb

**Figure 7:**
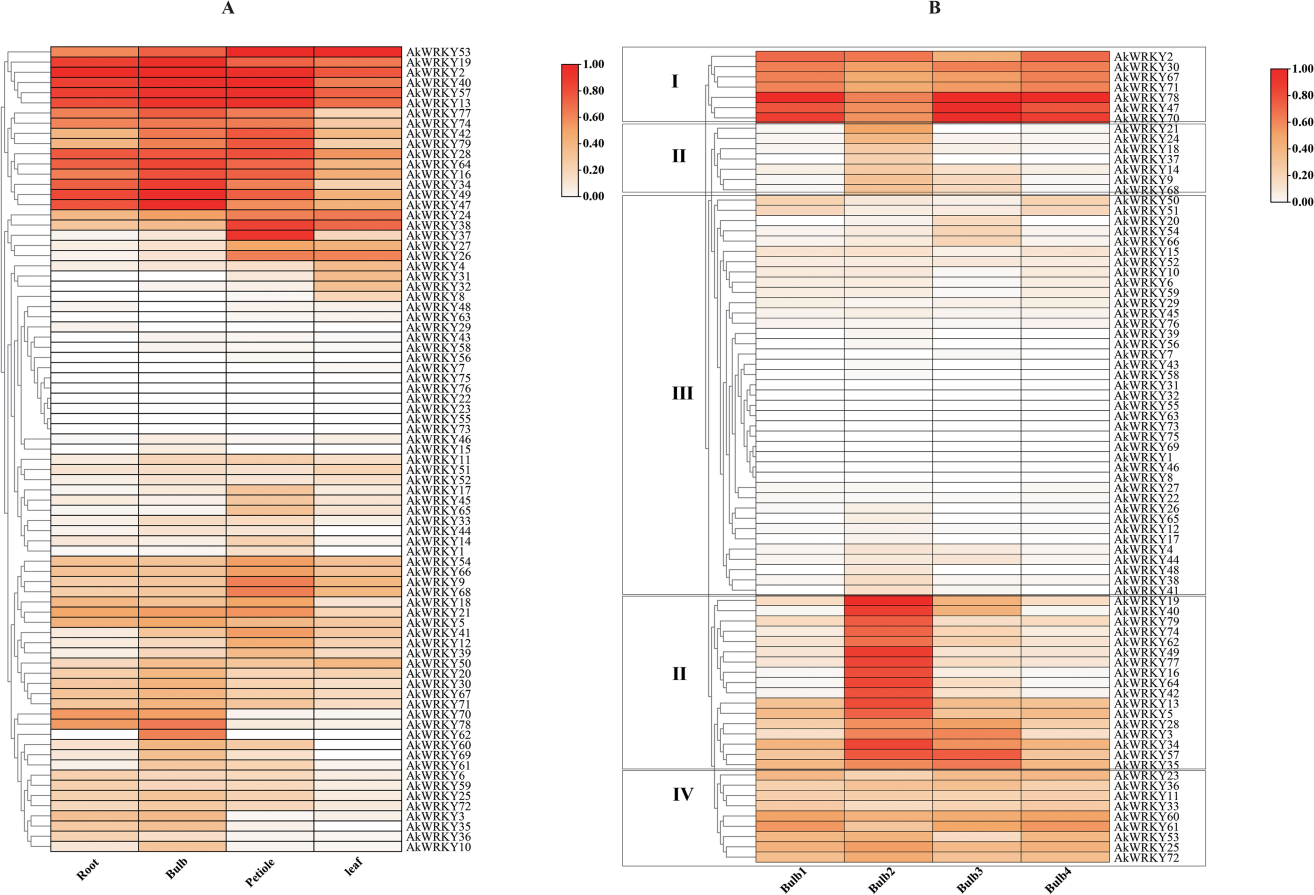
Heatmap of AkWRKY gene family expression profiles. **(A)** Expression of *AkWRKY* genes in different tissues, **(B)** Expression of *AkWRKY* genes across four corm developmental stages. Corm1 represents the dormancy stage, corms2 represents the “changing head” stage, corm3 represents the corms expansion stage, and corms represents the maturity stage, circle the different groupings with a black border.

To investigate tissue-specific expression of the *WRKY* family of *A. konjac*, the expression patterns of 79 *AkWRKYs* were analyzed in the root, corm, petiole, and leaf blades (PRJNA608095). *AkWRKY2* revealed relatively higher expression than other genes in both the root and the corm. *AkWRKY40* was specifically expressed at high levels in the petiole, and *AkWRKY50* exhibited the highest expression in the leaves (**Figure 7A**). Six genes—*AkWRKY2*, *13*, *19*, *40*, *53*, and *57*—were highly expressed across all four tissues of *A. konjac* (**Figure 7A**).

In this study, we also obtained transcriptome data from *A. konjac* corms at four developmental stages (PRJNA734512) (Gao Y. et al., 2022) and analyzed the expression patterns of *AkWRKYs* throughout corm development. The results indicated that the expression levels of *WRKY* family genes varied significantly across the different stages, indicating that these genes play different roles in the growth and development of the corm. Based on their expression patterns, the 79 *AkWRKYs* were categorized into four groups: Group I comprised seven genes, including *AkWRKY2*, whose expression first decreased and then increased during corm development. Group II, which comprised *AkWRKY21*, *19*, and 24 additional genes, exhibited peak expression during the second developmental stage of the corm (the “head changing stage”). Group III, represented by *AkWRKY50* and other genes, displayed consistently low expression across all developmental stages. In Group IV, nine genes—including *AkWRKY23*—were expressed across all four developmental stages of the corm. Among them, *AkWRKY78* displayed the highest expression in stages 1 (dormancy), 3 (corm expansion), and 4 (maturity), while *AkWRKY19* exhibited the highest expression in stage 2 (the “head changing” stage). Using stage 1 (dormancy) as the CK, most genes exhibited relatively higher expression levels in stages 2 and 3 compared to stage 4 (maturity) (**Figure 7B**).

### 3.8 qRT-PCR expression pattern analysis of *AkWRKYs*

**Figure 8:**
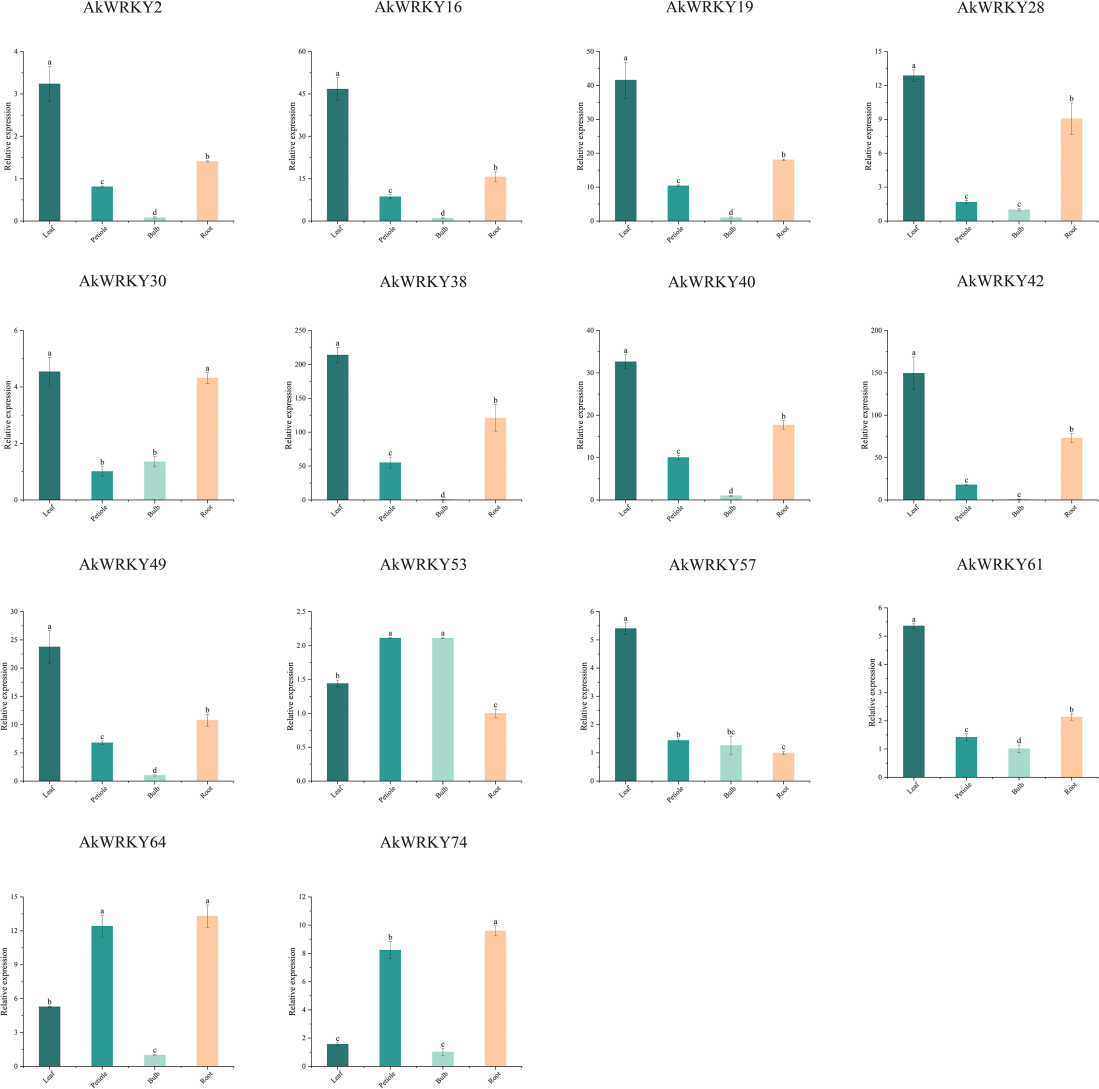
The qRT-PCR results of *AkWRKY* gene family members in different tissues. Note: mean value ± SE are shown for the 3 replicates. a, b, c, and d represent significance analysis, measured by different lowercase letters within a column according to the least significant different test (P<0.05).

Based on the transcriptome data and cis-acting element analysis results, a total of 14 genes—*AkWRKY2*, *16*, *19*, *28*, *38*, *40*, *42*, *49*, *53*, *57*, *64*, *74*, *30*, and *61*—were screened for qRT-PCR analysis. The *AkWRKYs* indicated tissue-specific expression across different organs (**Figure 8**). Ten genes exhibited the highest expression in leaves, significantly exceeding expression levels in the other three tissues. *AkWRKY30* displayed significantly higher expression in leaves and roots. *AkWRKY53* was most highly expressed in petioles and corm, differing significantly from its expression in other tissues. *AkWRKY64* displayed elevated expression in petioles and roots, and *AkWRKY74* revealed the highest expression overall.

**Figure 9:**
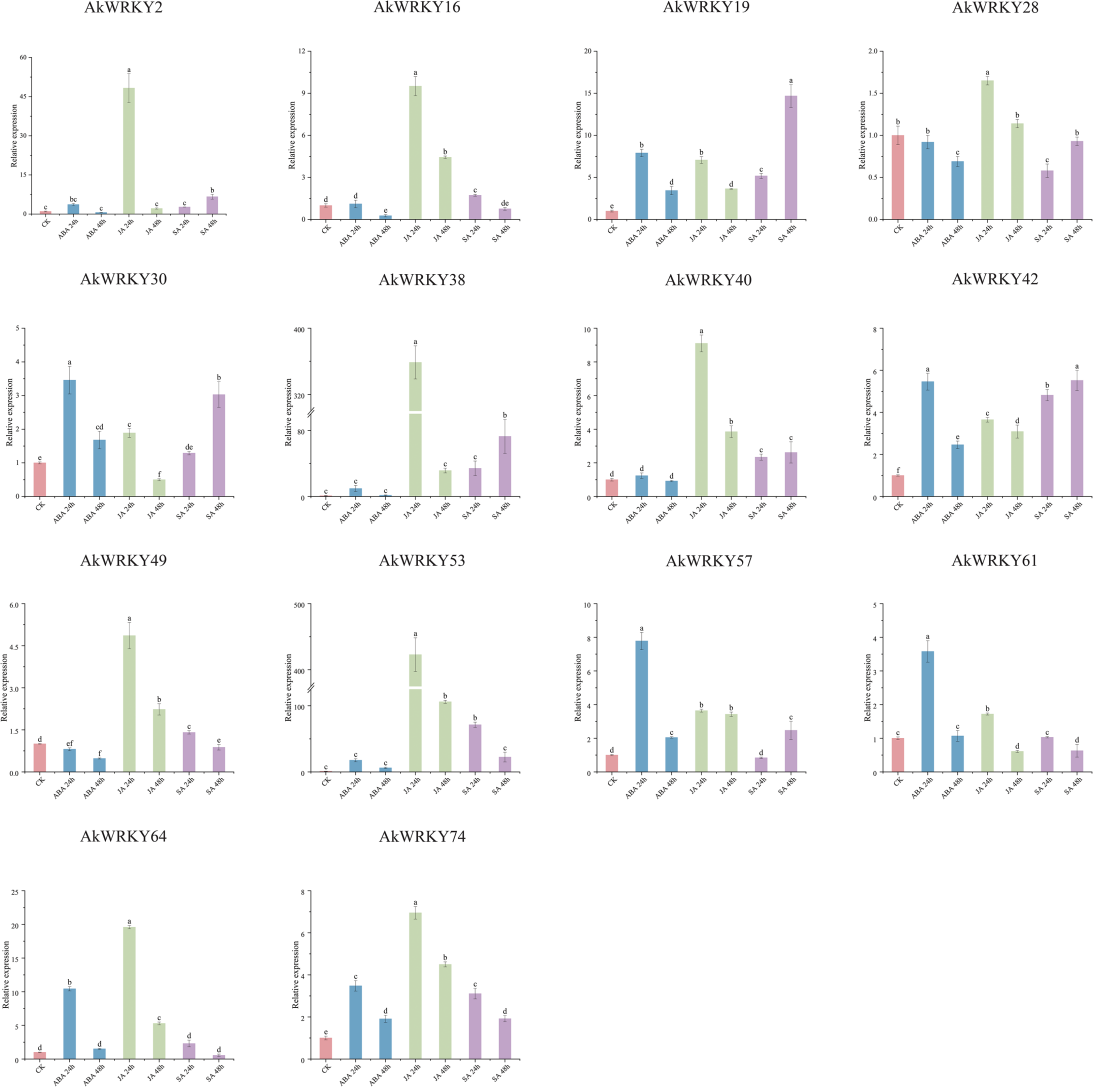
The qRT-PCR results of *AkWRKY* gene family members under different hormone treatments in A. konjac. Abbreviations: CK, Control group; ABA, Abscisic acid; JA, Jasmonic acid; SA, Salicylic acid. Note: mean value ± SE are shown for the 3 replicates. a, b, c, d, and e represent significance analysis, measured by different lowercase letters within a column according to the least significant different test (*P*<0.05).

This experiment also analyzed the response of 14 *AkWRKYs* to ABA, JA and SA treatments for 24 h and 48 h, respectively (**Figure 9**). *AkWRKY2*, *19*, *30*, *42*, *57*, *61*, *64*, and *74* were up-regulated significantly after 24 h of ABA treatment, with expression levels significantly higher than that of the CK and the 48-h treatment. No significant induction was observed for *AkWRKY16*, *38*, *40*, *53*, and *74*, with their expression levels remaining at basal levels comparable to the CK. In contrast, JA treatment for 24 h sharply induced the expression of *AkWRKY2*, *16*, *28*, *38*, *40*, *49*, *53*, *64*, and *74*, which was significantly more pronounced than the responses elicited by any other hormone tested. However, *AkWRKY19* exhibited significantly higher expression after 48 h of SA treatment compared to the CK and the other hormone treatments. *AkWRKY42* expression was significantly higher than that of the CK and the other hormone treatments across all three hormones—ABA, JA, and SA—at different time points. Its expression peaked at 24 h under ABA treatment and 48 h under SA treatment, with significant differences compared with the other hormone treatments. In addition, the expression of *AkWRKY16*, *49*, *53*, *61*, *64*, and *74* was higher under 24-h treatments of ABA, JA, and SA than under their respective 48-h treatments.

**Figure 10:**
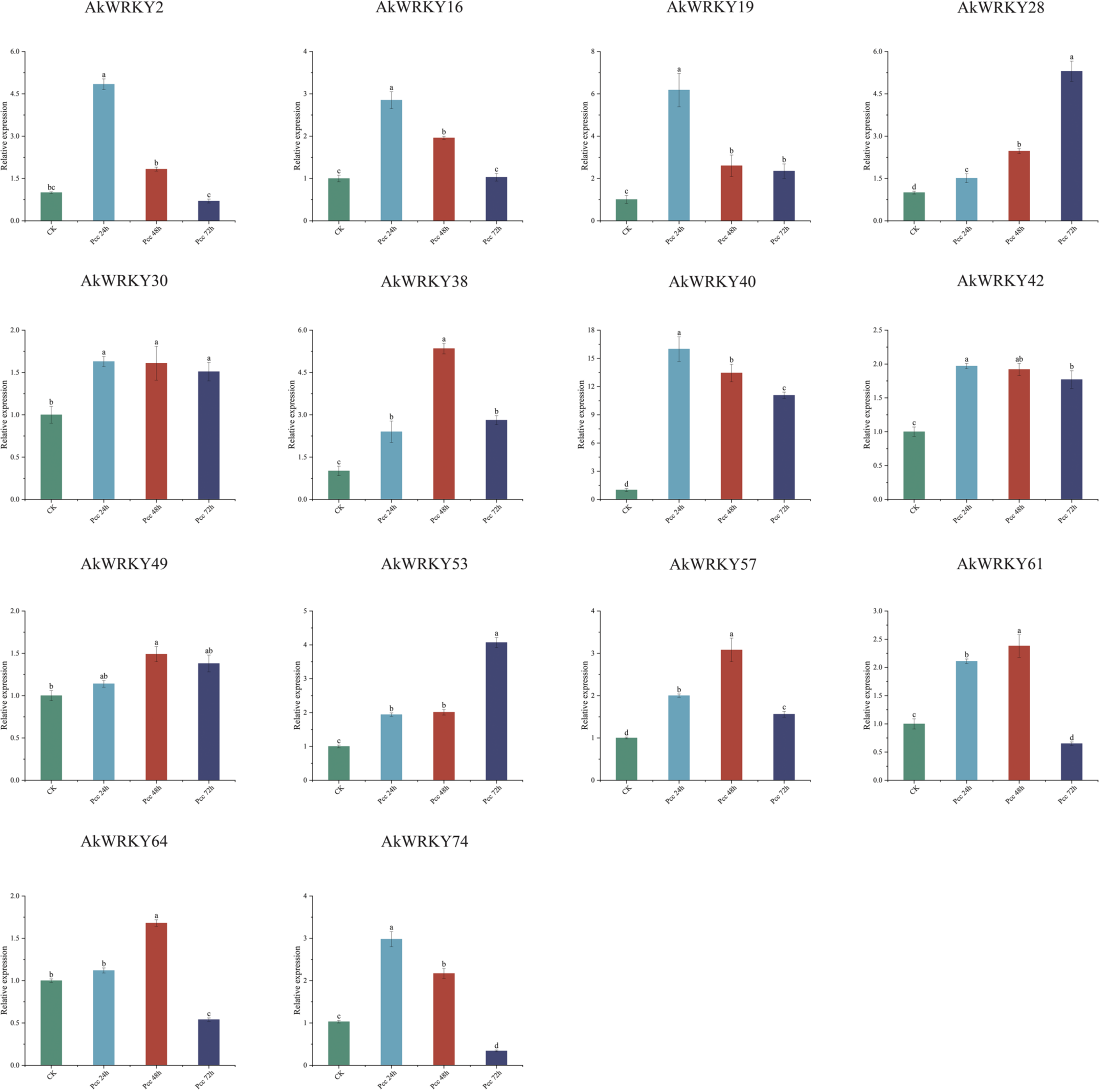
The qRT-PCR results of *AkWRKY* gene family members under biotic stress in *A. konjac*. Mean value ± SE are shown for the 3 replicates. a, b, c, and d represent significance analysis, measured by different lowercase letters within a column according to the least significant different test (*P*<0.05).

In the expression profile of *A. konjac* in response to *Pcc* infestation, 14 *AkWWRKYs* revealed temporal and spatial expression specificity (**Figure 10**). *AkWRKY2*, *16*, *19*, *40*, and *74* were significantly higher at the early stage (24 h) of *Pcc* infestation compared with the CK and the other infestation phases. *AkWRKY38*, *57*, *61*, and *64* were significantly higher at 48 h compared with CK and the other time treatments. The expression of *AkWRKY28* and *53* was significantly higher at 72 h than at 24 h, 48 h, and CK. *AkWRKY49* exhibited the highest expression at 48 h, although the difference was statistically non-significant compared with 24 h and 72 h. *AkWRKY30* displayed the highest expression at all three infestation time points, with no significant differences among them. Similarly, *AkWRKY42* was highly expressed across all time points, but its expression at 24 h was significantly higher than that at 72 h, whereas the expression at 48 h was not significantly different from that at 72 h.

**Figure 11:**
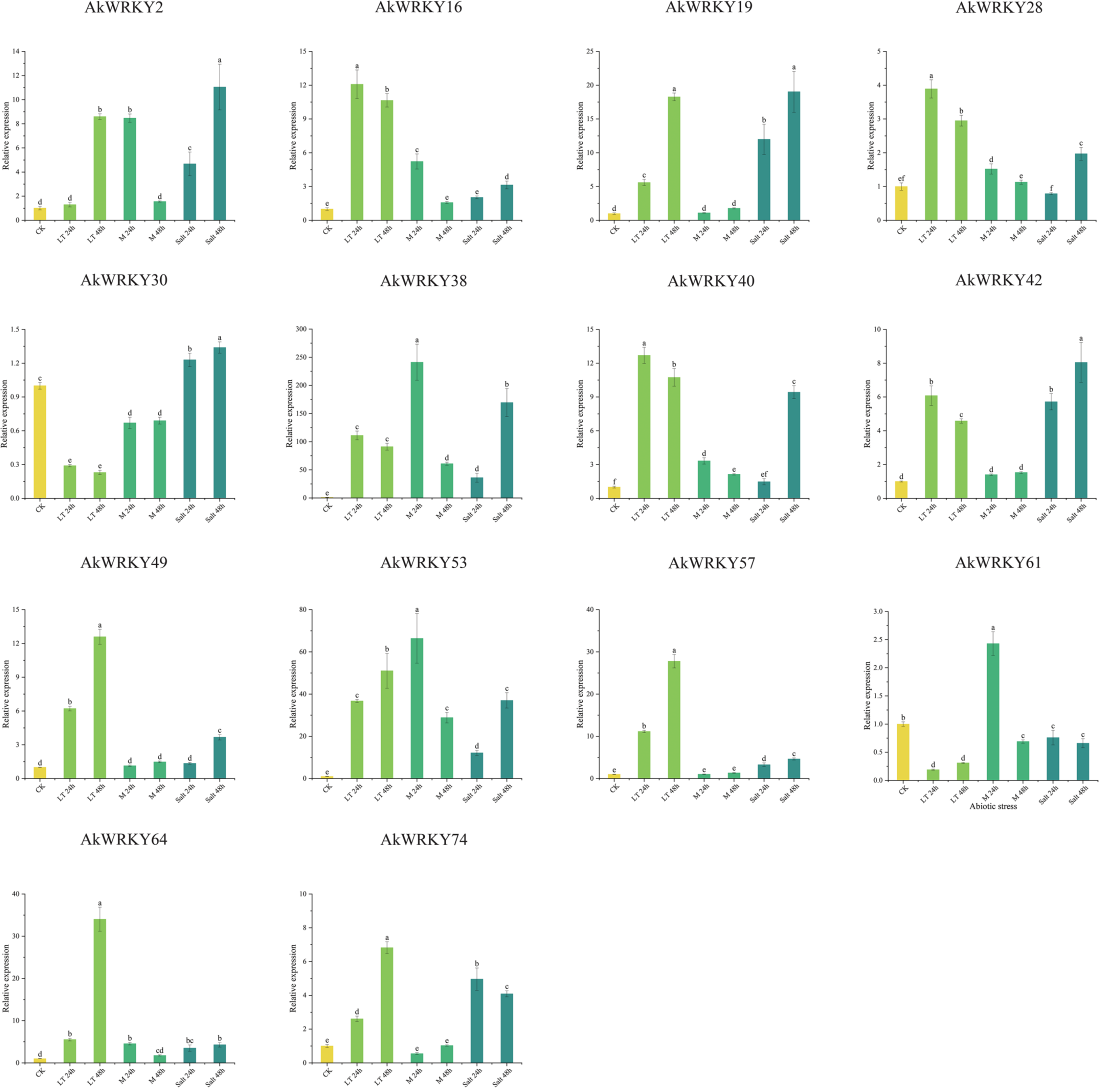
The qRT-PCR results of *AkWRKY* gene family members under abiotic stress in *A. konjac*. “ LT “ stands for low-temperature treatment, “ M “ stands for mannitol-mimicking drought treatment, and “Salt “ is salt treatment. Mean value ± SE are shown for the 3 replicates. Note: a, b, c, d, e, and f represent significance analysis, measured by different lowercase letters within a column according to the least significant different test (*P*<0.05).

To investigate the expression pattern of *AkWRKYs* under abiotic stresses, their expression levels were analyzed under low temperature, mannitol-mimicking drought, and salt stresses (**Figure 11**). Under low-temperature stress, the expression of *AkWRKY16*, *19*, *28*, *38*, *40*, *42*, *49*, *53*, *57*, *64*, and *74* was significantly higher than that in the CK. Among these, the expression of *28*, *38*, *40*, and *42* peaked at 24 h, whereas *AkWRKY2*, *19*, *49*, *53*, *57*, *64*, and *74* reached their highest expression at 48 h. The expression of *AkWRKY2* at 48 h was significantly higher than that at 24 h, although its expression at 24 h was not significantly different. In contrast, the expression of *AkWRKY16*, *19*, *28*, *38*, *40*, *42*, *49*, *53*, *57*, *64*, and *74* was significantly higher than that in the CK under low-temperature stress. Meanwhile, the expression of *AkWRKY30* and *61* was significantly lower in the CK, suggesting that these two genes may not be involved in the *A. konjac* response to low-temperature stress.

Under drought stress, the expression of *AkWRKY38*, *40*, and *53* was significantly higher than that in the CK at all periods, with peak expression observed at 24 h. This suggests that these genes may be involved in the drought response of *A. konjac*, particularly during the early phase of stress. The expression of *AkWRKY2*, *16*, *28*, *61*, and *64* under drought stress at 24 h was significantly higher than that under CK conditions and at 48 h. However, no significant differences were found between these genes and the CK at later time points. In contrast, the expression of *AkWRKY19*, *30*, *42*, *49*, *57*, and *74* revealed no significant changes compared to the CK at any time point during low-temperature treatment, except for *AkWRKY30*, which was significantly down-regulated (**Figure 11**).

Under salt treatment, the expression of *AkWRKY2, 19*, *30*, *38*, *42*, *53*, *57*, *64*, and *74* was significantly higher than that of the CK at 24 h and 48 h. The expression of six genes (*AkWRKY2*, *19*, *30*, *38*, *42*, *53*, and *57*) was significantly higher at 48 h than at 24 h, whereas the expression of *AkWRKY64* did not differ between 24 h and 48 h. *AkWRKY64* expression did not differ from the CK at different time points under low-temperature treatment, except for *AkWRKY30*, which was significantly lower than that of the CK (**Figure 11**). *AkWRKY64* expression was significantly higher at 48 h than at 24 h, suggesting that these genes may be involved in the response of *A. konjac* to salt stress, although the timing of the response varied. Expression of *AkWRKY16*, *28*, *40*, and *49* remained unchanged at basal levels (comparable to the CK) at 24 h, but a marked induction was observed at 48 h, with levels significantly exceeding both the earlier time point and the control. *AkWRKY61* expression was significantly lower than that of the CK at both 24 and 48 h of salt stress, suggesting that this gene may not be related to the response of *A. konjac* to salt stress. Overall, *AkWRKY38* and *53* were highly expressed under various abiotic stresses at different treatment time points (**Figure 11**).

## 4 Discussion

As one of the largest families of TFs, WRKYs are involved in various biological processes and have been extensively studied in many species, but rarely in Araceae (Dong et al., 2003; Chen et al., 2020). In this study, genome-wide identification, bioinformatics analysis, phylogenetic analysis, and protein function prediction were performed in four species of Araceae. In addition, the expression of *WRKYs* in *A. konjac* was analyzed, with a focus on the expression patterns of *AkWRKYs* under biotic and abiotic stresses. Previous related studies have demonstrated that there is no obvious positive correlation between the number of *WRKY* family members and genome size. For example, in oilseed crops, *Helianthus annuus* L. has the largest genome (3.60 Gb) but contains only 119 *WRKYs*, whereas *Brassica napus* L., with a smaller genome (1.13 Gb), possesses 283 *WRKYs* (Liu et al., 2020). Similarly, *Dimocarpus longan* Lour. (471.90 Mb) contains 55 *WRKYs*, which is fewer than the 72 found in *A. thaliana* (116.00 Mb) (Jue et al., 2018). In this study, *Z. elliottiana* exhibited 59 *WRKY* family members, slightly more than *A. albus*, which displayed 57. However, the genome size of *Z. elliottiana* (1.07 Gb) is less than one-fifth that of *A. albus* (5.59 Gb). These findings further support the conclusion that there was no clear correlation between genome size and the number of *WRKY* family members in plants (Xu et al., 2023).

Group II WRKY TFs are relatively abundant in plants and exhibit high evolutionary diversity, which can lead to greater environmental adaptability (Goyal et al., 2023). In this study, 231 *WRKY* members from Araceae were categorized into three groups—I, II, and III—with Group II having both the highest number of members. In contrast, *S. intermedia* showed consistently lower gene numbers across all groups, with an especially notable reduction in group III (only 1 gene). This observation corresponds with documented patterns of repeated *WRKY* gene family loss events in aquatic plants, particularly pronounced in group III members (Zhao et al., 2021). Previous research indicates that aquatic plants generally have contracted coding genes compared to terrestrial plants. These contracted gene families are functionally related to organ development, structural support, drought response, hormone regulation, and microbial defense (Guo et al., 2025). Studies have shown that transcripts of the group IIa genes *WRKY62* and *WRKY76* accumulate in response to benzothiadiazole, SA, and the rice fungal pathogen *Magnaporthe grisea*, while the expression of *WRKY71* is induced by SA and bacterial pathogen infection in rice. Furthermore, when *WRKY62*, *WRKY28*, *WRKY71*, and *WRKY76* are simultaneously overexpressed, these four genes interact through complex functional mechanisms—potentially via the formation of protein complexes—to enhance rice basal resistance against *Xanthomonas oryzae* pv. *oryzae* (*Xoo*). Given these findings, it is plausible that the *AkWRKY16*, *26*, *27*, *55*, *56*, and *64* genes from *A. konjac*, which also belong to group IIa, may employ a similar regulatory mechanism in response to pathogen stress (Peng et al., 2010). *AtWRKY25* and *26*, located in subfamily I, act as positive regulators under both heat and cold stress, whereas *AtWRKY33* is rapidly induced only under cold stress (Fu and Yu, 2010). *AtWRKY45* and *75*, both belonging to subfamily IIc, are involved in *A. thaliana*’s acclimatization to low-phosphorus stress by regulating genes associated with root morphology and phosphorus uptake, thereby enhancing tolerance (Deng et al., 2023). *AtWRKY11* (IId) and *70* (III) coordinate resistance to *Bacillus* through JA and SA signaling pathways; their overexpression enhances drought tolerance and promotes seed germination and root growth in *A. thaliana* (Song et al., 2023). Members of the *WRKY* family in Araceae that belong to the same subfamily may perform similar biological functions (Liu et al., 2023a).

Cis-acting elements play an important role in gene transcription and expression. Several types of cis-acting elements were identified in the promoters of *WRKYs* from the four species in this study, including hormone response elements (ABRE, AuxRR-core, CGTCA-motif, GARE-motif, P-box, TCA-element, and TGA-element), plant physiological metabolism-related elements (A-box, CAAT-box, CAT-box, CCAAT-box, MSA-like, O2-site, and TATA-box), and stress response elements (ARE, GC-motif, LTR, MBS, TC-rich repeats, and WUN-motif). Among these, ABRE, CGTCA-motif, CAAT-box, and ARE were high-frequency elements in most *WRKY* promoters. At least one hormone-responsive element and one stress-responsive element were present in each *WRKY* promoter, suggesting that *WRKYs* in the four Araceae species studied may be involved in both hormone and stress responses. Drought stress in plants causes an increase in ABA levels (Brookbank et al., 2021), and *WRKYs* can be rapidly induced, triggering a signaling network that ultimately enhances plant stress tolerance (Jiang et al., 2017). The promoter regions of the vast majority of members in this study contained ABRE and MBS elements, suggesting that most may be involved in drought response through an ABA-dependent pathway (Zhu et al., 2022). In this study, compared with the control group, the expression levels of genes *AkWRKY19*, *30*, *38*, *42*, *53*, *57*, and *61* were relatively higher at 24 and 48 hours after ABA treatment. Similarly, the expression levels of genes *AkWRKY2*, *16*, *28*, *38*, *40*, and *53* were relatively higher than those in the control group at 24 and 48 hours under drought treatment (**Figure 10**). The promoter of the aquatic plant *Spirodela polyrhiza* is enriched with a large number of anaerobically induced ARE elements (Zhao et al., 2021), suggesting that the evolutionary divergence of *WRKY* promoter sequences may be affected by different aquatic plant habitats. For example, the content of LTR elements in *WRKY* promoters of submerged aquatic plants is relatively low due to the relatively stable water temperature (Guo et al., 2025). In this study, the abundance of ARE and GC motifs in the *WRKY* promoter of the aquatic plant puffball further supports the idea that WRKY TFs may act as key regulators of the hypoxic stress response triggered by frequent submergence in aquatic environments.

In this study, 13, 18, 24, and 11 pairs of segmental duplication genes were found in *A. konjac* (**Figure 5**), *A. albus*, *Z. elliottiana*, and *S. intermedia* (**Supplementary Figure S3**), respectively, suggesting that segmental duplication is the major mode of *WRKY* family expansion in Araceae, similar to what has been observed in the Chinese Rose (*Rosa chinensis*) *WRKY* family (Yan et al., 2024). A total of 65 pairs of segmental duplicated genes in the four species displayed Ka/Ks ratios lower than one, demonstrating that these genes may have undergone purifying selection, which constrains non-synonymous mutations to maintain functional stability during their evolutionary history (Wang et al., 2022). However, the *SiWRKY17* and *21* pair revealed only synonymous mutation sites, which may be attributed to their large sequence differences and long evolutionary distance (**Supplementary Figure S3C**) (Huang et al., 2021). The variation in the number of colinear gene pairs between *A. konjac* and the other three Araceae species is likely related to the degree of evolutionary relatedness among the species (Feng et al., 2023).

Based on the function of WRKY proteins in *A. thaliana*, the potential regulation of WRKY proteins in the Araceae family can be predicted, including gene function and its relationship with overall biological processes. In this study, AkWRKY proteins demonstrated high sequence similarity with *AtWRKY1*, *33* (SIB1/SIB2), *60*, and *63*. It has been demonstrated that *AtWRKY1*, *60*, and *63* all interact specifically with common W-box inducer response elements, suggesting that *AkWRKYs* may be involved in plant responses to biotic or abiotic stresses and may mediate stress adaptation by regulating downstream gene expression (Liu J. et al., 2024). *AtWRKY33* (SIB1/SIB2) mediates stress adaptation by regulating chloroplast metabolism to enhance defense and plays a vital role in plant resistance to pathogen infection (Zheng et al., 2006) (**Supplementary Figure S5**). It is hypothesized that *AkWRKY* proteins may respond positively under pathogen infestation. Maximum-likelihood analysis showed that *AkWRKY19* and *AkWRKY49* formed a monophyletic group with *AtWRKY33* (**Figure 2**), suggesting they may be functional orthologs. Furthermore, our study revealed that both *AkWRKY19* and *AkWRKY49* were significantly upregulated at 24 h, 48 h, and 72 h post-*Pcc* infection, with *AkWRKY19* peaking at 24 h and *AkWRKY49* reaching its maximum expression level at 48 h. As close phylogenetic homologs of *AtWRKY33*, these findings strongly suggest their potential involvement in defense responses of *A. konjac*. VQ20 contains proteins with VQ motifs, which may act as negative regulators of plant defense (Shaik and Ramakrishna, 2013). TIFY 5A (AT1G30135) and TIFY 6A (AT1G48500) act as JA signaling pathway repressors that negatively regulate plant responses to JA (Liu et al., 2023b), suggesting that AkWRKY proteins may play a regulatory role in JA-mediated defense responses (for example, disease and insect resistance) and growth and development (for example, pollen development and fruit ripening) (He et al., 2020). The above results suggest that *A. konjac* WRKY proteins play a major role in hormone regulation, plant growth and development, and adversity stress (Hu et al., 2021). Through GO and KEGG functional annotation, it was observed that AkWRKY proteins may regulate environmental adaptation, signal transduction, and gene expression processes in plants by regulating transcription, participating in plant–pathogen interactions, the MAPK signaling pathway, and other key pathways. They may play important roles in plant metabolism, growth, and development, and responses to biotic stresses (for example, pathogen infections), and abiotic stresses (for example, environmental stimuli) in *A. konjac* plants (Ren et al., 2021; Chen Y. et al., 2023).

It was demonstrated that members of the *WRKY* family are tissue-specific. For example, in safflower (*Carthamus tinctorius* L.), four genes—*CtWRKY17*, *22*, *25*, and *49*—were preferentially expressed in leaves, flowers, and roots, and four genes—*CtWRKY11*, *34*, *35*, and *82*—were highly expressed in different tissues under EBR and light stress (Liu et al., 2020). In Purple Falsebrome (*Brachypodium distachyon*), *BdWRKY78* was highly expressed in roots, while its expression in leaves and stems was relatively low. *BdWRKY32*, *41*, *73*, and *74* were expressed at higher levels in stems than in leaves and roots (Wen et al., 2020). In wild potato (*Solanum commersonii*), *ScWRKY002*, *013*, and *017* were highly expressed only in flowers, whereas *ScWRKY042* and *080* were highly expressed only in leaves (Yuan et al., 2021). The expression of the vast majority of genes in the petiole in this study was significantly higher than that in the other three tissue organs, probably because the petiole plays key roles in *A. konjac* (pathogen defense, high-intensity support, and nutrient transport). These specific functions may induce a large number of *WRKYs* to respond with preferential and high-level transcriptional activation in petiole tissues (**Figure 8**) (Hejnowicz, 2005). This further validates the observation that WRKY TFs exhibit different expression profiles across various organs or tissues.

Because a large number of cis-acting elements related to plant growth and development, hormonal signaling, and stress responses are enriched upstream of the *AkWRKY* promoter, we analyzed their expression patterns using qRT-PCR. Specifically, we analyzed the expression of *AkWRKYs* under different hormone treatments, as well as biotic and abiotic stress conditions.

ABA induces the expression of relevant genes in stomatal defense cells, reduces gas exchange between plants and the external environment, and decreases water loss, thereby alleviating damage caused by high salt, drought, or low-temperature stresses (Garbeva and Weisskopf, 2020). JA can transmit signals to induce the expression of plant defense genes when plants are subjected to stress (Båga et al., 2022). SA is an endogenous plant signaling molecule that plays an important role in the hypersensitive response when attacked by pathogenic bacteria and in the development of systemic acquired resistance (Wu et al., 2016). In *Limonium bicolor*, the expression of the *LbWRKY10* gene was significantly elevated under ABA treatment, and silencing of this gene reduced salt gland density and salt tolerance (Zhu et al., 2024). In *Scutellaria baicalensis* Georgi, the expression of the *SbWRKY41* was up-regulated 40-fold under JA stress (1 h and 24 h). The expression of *SbWRKY41* and *62* was increased 20-fold after 24 h of ABA treatment (Zhang et al., 2022). In *Glycine max*, most genes, such as *GmVQ2*, *29*, and *69*, were up-regulated under SA treatment (Wang et al., 2019). In this study, 14 *AkWRKYs* revealed differential responses to ABA, JA, and SA treatments. Among them, several genes—*AkWRKY2*, *16*, *28*, *38*, *40*, *49*, *53*, *64*, and *74*—were significantly up-regulated under JA hormone treatment (**Figure 9**). These results suggest that *AkWRKYs* may be widely involved in the positive response of *A. konjac* to biotic and abiotic stresses.

Currently, many *WRKYs* also play significant roles in biotic stress responses. For example, in *Nicotiana attenuata*, *NaWRKY70* regulates capsidiol biosynthesis, a key antimicrobial compound, and its silencing results in reduced ABA production and compromised pathogen defense (Song and Wu, 2024). In *Solanum tuberosum*, *StWRKY8* is involved in the biosynthesis of benzylisoquinoline alkaloids, which possess antimicrobial activity and contribute to cell wall reinforcement, thereby limiting pathogen spread (Yogendra et al., 2017). The expression pattern of *A. konjac WRKYs* under biotic stress in this study revealed that the expression trends of 14 *AkWRKYs* under *Pcc* treatment for 24 h, 48 h, and 72 h corresponded to the dynamics of the pathogen infestation process and the *A. konjac* defense response. This may be because *A. konjac* initiated a series of early (24 h) defense responses, such as cell wall reinforcement, production of antioxidant substances, and synthesis of antimicrobial substances (Buerstmayr et al., 2021). With continued pathogen infestation (48 h), the pathogen may have secreted effectors that interfere with the defense signaling pathway of *A. konjac* (Fu et al., 2022). The immune response of *A. konjac* may then enter a regulatory adjustment phase, accompanied by a decrease in the expression of *AkWRKY2*, *16*, *19*, *28*, *30*, *40*, *42*, *53*, and *74* (Zhao et al., 2024). By 72 h, the *AkWRKY28* and *53* may be involved in signaling for acquired resistance, and thus their expression rises again, allowing *A. konjac* to acquire greater resistance to *Pcc* (**Figure 10**) (Divya et al., 2018). This may also be due to different synergistic or antagonistic effects of phytohormone synthesis and signaling occurring at various stages of *Pcc* infestation, which in turn affect the expression of *AkWRKYs* (Wang et al., 2015).

When plants sense stress, the corresponding signals are activated and transferred to the cell interior. In response to abiotic stress, some WRKY TFs can be rapidly and differentially expressed to promote signaling and regulate the expression of related genes (Jiang et al., 2017). For example, the expression of *ZmWRKY40* (*Zea mays* L.) (Wang et al., 2018), *ScWRKY5* (*Saccharum officinarum* L.) (Wang et al., 2020), and *PbrWRKY5*3 (*Pyrus betulifolia* Bunge) (Liu et al., 2019) was induced and up-regulated under drought and salt stress. In this study, the expression of *AkWRKY16*, *28*, *40*, *49*, *57*, *64*, and *74* was relatively higher under low-temperature stress compared with other treatments. The expression of *AkWRKY16*, *28*, and *40* was also relatively higher under drought stress, whereas *AkWRKY2*, *19*, *30*, and *42* indicated higher expression levels under salt stress compared to the CK and the other two treatments (**Figure 11**). The results suggest that these genes may play a positive regulatory role in the response of *A. konjac* to low temperature, drought, and salt stress (Sun et al., 2021).

## 5 Conclusion

In this study, the *WRKY* TF families of four Araceae species were identified and functionally characterized genome-wide for the first time. Through systematic analysis of the *WRKY* families of *A. konjac*, *A. albus*, *Z. elliottiana*, and *S. intermedia*, a total of 231 members were identified, with the highest proportion belonging to class II structural domain members (88.31%). Conserved motif and gene structure analyses revealed that members of the same subgroup were highly conserved, whereas significant differences were observed among different subgroups. Species evolutionary analyses indicated that the expansion of the *WRKYs* family in Araceae was mainly driven by segmental duplication events, and the Ka/Ks ratios (all < 1) supported the predominant role of purifying selection in gene retention. Promoter cis-acting element analysis revealed that all Araceae *WRKYs* contained hormone-responsive elements (for example, ABRE and CGTCA-motif) and stress response elements (for example, MBS and TC-rich repeats). Interaction network analysis further confirmed that core nodes such as *AkWRKY33* and *60* were closely associated with plant disease resistance and metabolic regulatory pathways. qRT-PCR analysis revealed that *AkWRKYs* exhibited significant tissue specificity. qRT-PCR verified the expression of 14 candidate genes under ABA, JA, and SA hormone treatments, low-temperature, drought, and salt stress treatments, as well as during the dynamic response to *Pcc* infestation. *AkWRKY30*, *42*, and *57* displayed high expression under ABA treatment. *AkWRKY2*, *38*, *53*, and *64* revealed high expression under JA treatment, and *AkWRKY19* and *20* exhibited high expression under SA treatment. *AkWRKY16*, *40*, *49*, *57*, *64*, and *74* were highly expressed under low-temperature stress, while *AkWRKY53* was strongly expressed under drought stress. *AkWRKY2*, *19*, and *42* displayed high expression under salt stress, and *AkWRKY40* was highly expressed in response to *Pcc* infestation. Among these, *AkWRKY40* and *42* exhibited high expression under both biotic and abiotic stresses. These genes may serve as key targets for breeding stress-tolerant *A. konjac* varieties. This study not only provides new insights into the functional evolution of *WRKYs* in Araceae but also lays a theoretical foundation for the improvement of disease-resistant Araceae varieties and the analysis of secondary metabolism regulatory networks.

## Data Availability

The datasets presented in this study can be found in online repositories. The names of the repositories and accession numbers can be found in the article/**Supplementary Material**.

## Supplementary material

Supplementary Figure 1 Unrooted phylogenetic tree resulting from the comparative analysis of WRKY domains across the six species. This tree, which served as the basis for the rooted tree in **Figure 2**, is visualized here with the same clade-specific color scheme for clarity.

Supplementary Figure 2 Analysis of conserved motif characteristics in *WRKY* gene family members across four Araceae species: **(A)***A. thaliana*, **(B)***A. albus*, **(C)***Z. elliottiana*, and **(D)***S. intermedia*.

Supplementary Figure 3 UpSet plot of cis-regulatory elements in the 2000-bp upstream promoter regions of *WRKY* gene family members across four species: *A. konjac*, *A. albus*, *Z. elliottiana*, and *S. intermedia*.

Supplementary Figure 4 Collinearity relationships within *WRKY* genome across four species in the Araceae family, The colored lines in the middle represents the collinear relationship within the *WRKY* gene; The first circle represents the chromosome; The second circle represents the density of the gene (in heat map form): **(A)***AaWRKY* gene, **(B)***ZeWRKY* gene, **(C)***SiWRKY* gene.

Supplementary Figure 5 Protein interaction network for 79 AkWRKY proteins based on these orthologs in *A. thaliana*.

Supplementary Figure 6 Functional enrichment analysis of *AkWRKY* genes. **(A)** Gene ontology (GO) terms of *AkWRKY*. BP (Biological Processes), CC (Cellular Constituents), MF (Molecular Functionalities). **(B)** KEGG pathway analysis of *AkWRKY*.
