# Supplementary figures and images for: Genome-wide characterization of WRKY family genes in four araceae species and their expression analysis in Amorphophallus konjac
Source: Front Plant Sci. 2025 Dec 12;16:1671100. doi: 10.3389/fpls.2025.1671100 (PMC12741084; doi:10.3389/fpls.2025.1671100)

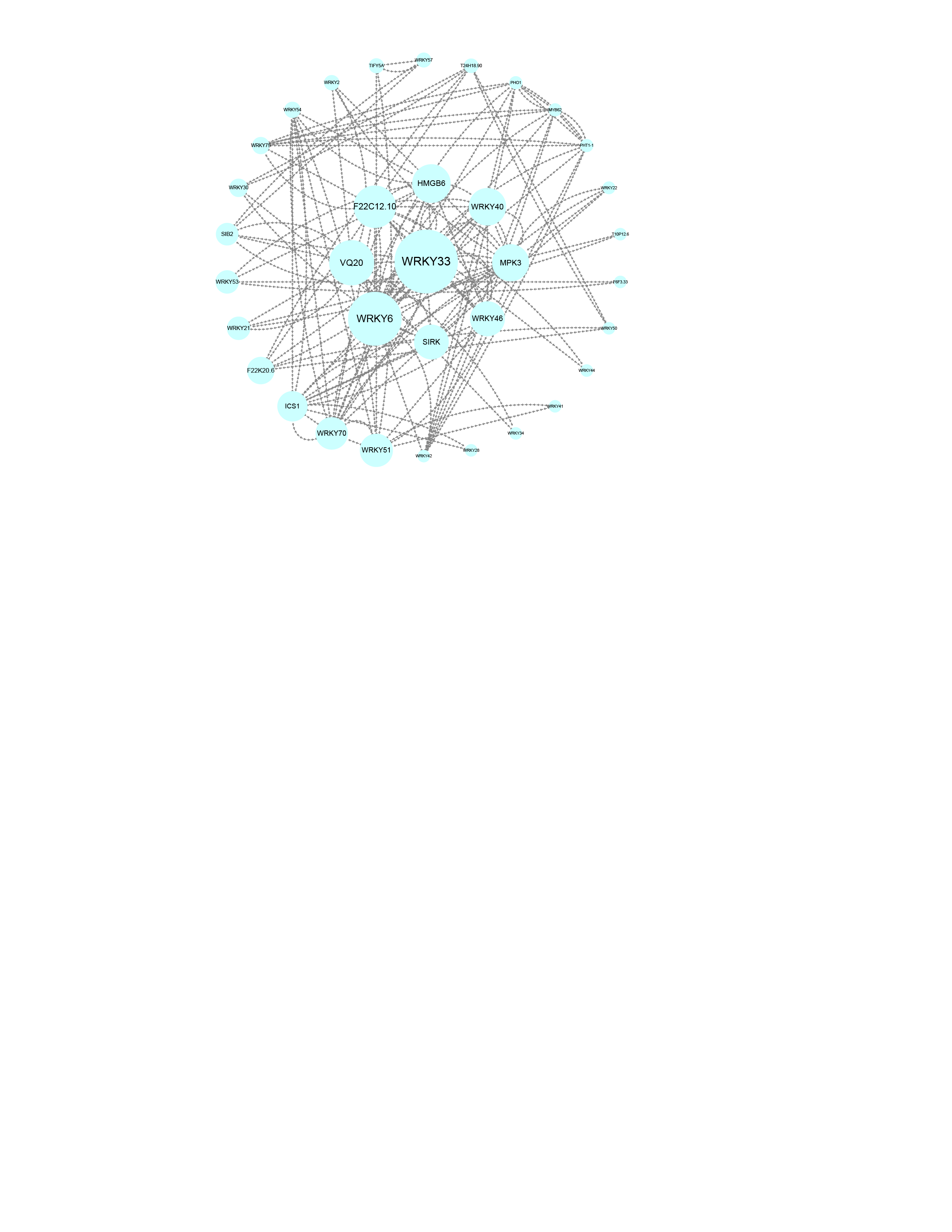

Supplement: Supplementary Figure 3 — UpSet plot of cis-regulatory elements in the 2000-bp upstream promoter regions of WRKY gene family members across four species: A. konjac, A. albus, Z. elliottiana, and S. intermedia. [file Image3.tif]

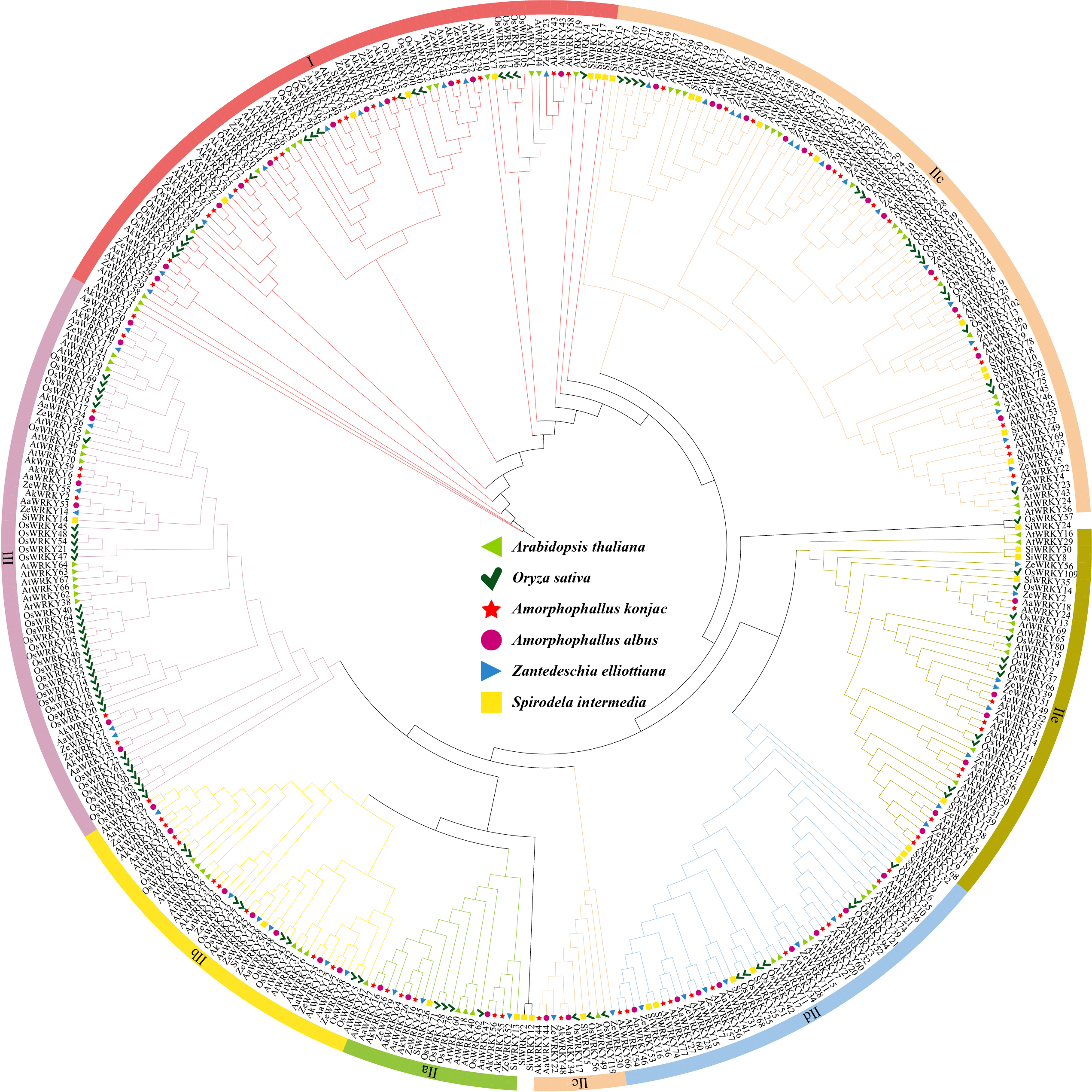

Supplement: Supplementary Figure 5 — Protein interaction network for 79 AkWRKY proteins based on these orthologs in A. thaliana. [file Image5.tif]
